# DNA methylation-based classification and identification of bladder cancer prognosis-associated subgroups

**DOI:** 10.1186/s12935-020-01345-1

**Published:** 2020-06-17

**Authors:** Zijian Tian, Lingfeng Meng, Xingbo Long, Tongxiang Diao, Maolin Hu, Miao Wang, Ming Liu, Jianye Wang

**Affiliations:** 1 Department of Urology, Beijing Hospital, National Center of Gerontology, Institute of Geriatric Medicine, Chinese Academy of Medical Sciences, No. 1 DaHua Road, Dong Dan, Beijing, 100730 China; 2 Graduate School of Peking Union Medical College, Chinese Academy of Medical Sciences, 9 DongDan SANTIAO, Beijing, 100730 China

**Keywords:** DNA Methylation, Urinary bladder neoplasms, Epigenetics, Cluster analysis

## Abstract

**Background:**

Bladder cancer (BCA) is the most common urinary tumor, but its pathogenesis is unclear, and the associated treatment strategy has rarely been updated. In recent years, a deeper understanding of tumor epigenetics has been gained, providing new opportunities for cancer detection and treatment.

**Methods:**

We identified prognostic methylation sites based on DNA methylation profiles of BCA in the TCGA database and constructed a specific prognostic subgroup.

**Results:**

Based on the consistent clustering of 402 CpGs, we identified seven subgroups that had a significant association with survival. The difference in DNA methylation levels was related to T stage, N stage, M stage, grade, sex, age, stage and prognosis. Finally, the prediction model was constructed using a Cox regression model and verified using the test dataset; the prognosis was consistent with that of the training set.

**Conclusions:**

The classification based on DNA methylation is closely related to the clinicopathological characteristics of BCA and determines the prognostic value of each epigenetic subtype. Therefore, our findings provide a basis for the development of DNA methylation subtype-specific therapeutic strategies for human bladder cancer.

## Background

Bladder cancer (BCA) is the ninth most common cancer in the world [1], and it is estimated that the number of new BCA cases in the United States will reach 81,400 in 2020, with approximately 17,980 deaths [2]. Notably, the clinical course of BCA varies with the degree of tumor invasion. In patients with BCA, 70% of cases are non-muscular invasive BCA (NMIBC), characterized by a high recurrence rate and low mortality. The remaining 30% are muscular invasive BCA (MIBC), which is prone to early metastasis, and approximately half of these cases are fatal [3, 4]. Cystoscopy is the current gold standard to monitor BCA. However, this method involves a highly invasive examination and has a sensitivity of 85% for the diagnosis of exophytic tumors [5]. It is worth noting that the postoperative pathology of transurethral bladder tumors does not always accurately reflect the tumor stage, due to limitations such as the experience of the operator and destruction of tissue caused by energy instruments. Studies have shown that the proportion of transurethral surgical procedures that underestimate tumor stage could be as high as 25% [6, 7]. Further, repeated cystoscopy is used for continuous follow-up, which makes BCA one of the most expensive malignant tumors to treat [5, 8]. At the same time, in the absence of a new targeted therapy-based strategy, despite surgery and cisplatin-based chemotherapy, it is clear that the existing treatment modality has reached its peak, with only a slight improvement in patient survival but more side effects [9]. As the survival time of patients with NMIBC progression to MIBC is poor [10], it is very important to better understand the biological pathogenesis of BCA, to more accurately monitor its progression, predict patient prognosis, and choose appropriate treatments.

Therefore, the existing diagnosis, prognosis, and monitoring systems for BCA patients need to be improved. Epigenetics comprise heritable but reversible modifications that can alter gene expression without changing the original DNA sequence. The maintenance of epigenome function is the basis of normal gene expression. Changes in epigenome function will affect the basic processes of cell proliferation, differentiation, and death, which might lead to cancer [11]. Therefore, the diagnosis, the evaluation of prognosis and the prediction of response to treatment can prospectively count on cancer biomarkers based on epigenetics [12]. DNA methylation was the first epigenetic modification found in humans in the early 1980s [13]. Among the epigenetic mechanisms, DNA methylation is the best studied, and abnormal CpG island methylation was proven to be related to the occurrence and development of many cancer types, including BCA [14]. At the same time, several studies also support this argument. Specifically, 90% sensitivity and 93% specificity were observed when evaluating the methylation of *Twist1* and *NID2* [15]. The methylation status of *SOX*-*1*, *IRAK3*, and *LI*-*MET* genes also showed better ability to predict progression than cystoscopy [16]. Uromark is described as a novel next-generation sequencing-based biomarker, based on 150 CpGs, with a sensitivity of 98% and a specificity of 97% for monitoring BCA [17]. Further, in terms of prognosis, hypermethylated *TIG1, GSTP1* and *APC* in BCA patients were found to be associated with poor survival outcomes, showing 93% specificity and 80% sensitivity [18].

However, it is recognized that the specific methylated sequence of the gene promoter region has not been identified yet. Therefore, the objective of this study was to identify DNA methylation profiles in BCA from the TCGA database and to identify biologically and clinically relevant molecular subsets. Our classification scheme could help to identify new BCA molecular subtypes and prognostic model based on methylation site to accurately subdivide BCA patients and improve clinical prognostic assessments and personalized treatment.

## Methods

### Data selection and pre-processing

For this study, Samples from the TCGA database containing 437 BCA methylation data are downloaded from UCSC Cancer Browser (http://xena.ucsc.edu/,2020-02-23). RNA-sequencing data from 433 BCA samples were downloaded from TCGA (https://cancergenome.nih.gov/, 2020-02-23), and among these, 407 samples were associated with clinical data. CpGs with missing data in more than 70% of the samples were excluded from the analysis. Based on the polymorphic CpGs and cross-reaction probe, the CpGs of the cross-reaction probe in the genome was also removed. The k-nearest neighbor imputation method in SVA R software package was used to estimate other unrecognized probes [19]. We also removed unstable genomic sites with CpGs and single nucleotide polymorphisms in sex chromosomes. We only studied the CpGs in the promoter region because DNA methylation in the promoter region (2 kb upstream of the transcriptional initiation site to 0.5 kb downstream) significantly affects gene expression. Finally, the BCA samples were randomly grouped as 203 training samples and 204 testing samples. The TNM staging system is based on the extent of the tumor (T), the extent of spread to the lymph nodes (N), and the presence of metastasis (M). For bladder cancer, T describes how far the main tumor has grown through the bladder wall and whether it has grown into nearby tissues. N indicates any cancer spread to lymph nodes near the bladder. M indicates if the cancer has spread to distant sites. Once a person’s T, N, and M categories have been determined, usually after surgery, this information is combined in a process called stage grouping to assign an overall stage. Stage grouping range from stages I through IV. Stage I to stage IV represents increasing malignant degree of bladder cancer, the earliest stage cancers are called stage I, and stage IV means advanced cancers.

### Using Cox proportional hazard regression model to determine CpGs of prognosis

First, a univariate Cox proportional hazard regression model was established based on CpGs, T stage, N stage, M stage, grade, age, sex, stage, and survival data (P < 0.001). The significant CpGs obtained from the univariate Cox proportional hazard regression model were used to analyze the multivariate Cox proportional hazard regression mode (P < 0.001). Finally, CpGs that were significantly modulated in multivariate Cox regression analyses were selected as characteristic CpGs.

### Establishment of molecular subtypes using consensus clustering

Based on the most variable CpG sites, the K-means clustering algorithm in ConcensusClusterPlus R packet [20] was used to perform consistent clustering to identification BCA subgroup. The pre-specified dataset was classified into k clusters via the algorithm. Here, 80% of the tumor samples were selected in each iteration, and the K-means algorithm and Euclidean square distance measure served as the grouping of Kappa 2 and 20. We used more than 100 iterations to determine the stability of each cluster. At the same time, the maximum number of clusters which have at least 90% cluster consensus was selected.

The optimal number of clusters was defined based on the delta area map and the cumulative distribution function. Color gradients were used to describe common values from 0 to 1 (white to dark blue). Items belonging to the same cluster in the matrix should be adjacent to each other. Therefore, the diagonal blue blocks on the white background in the color-coded heat map correspond to the matrix of perfect consensus. The best cluster number need to meet the following requirements: high consistency, small coefficient of variation, and the area under the cumulative distribution function (CDF) curve not increasing remarkably. The number of categories was defined by the relatively insignificant change in the area under the CDF curve. The heat map corresponding to consensus clustering was generated with the pheatmap R packet.

### Survival and clinical characteristics analyses

Construction of the overall survival (OS) curve of BCA subsets defined by the DNA methylation profile was performed via the Kaplan–Meier method. Significant differences among clusters were evaluated by the log-rank test. A comprehensive analysis of the relationship between clinical information and biological characteristics of clusters based on DNA methylation was performed through a Chi square test. R Bioconductor survival package was used for survival analysis. P < 0.05 was considered statistically significant.

### Gene Ontology enrichment and KEGG pathway analysis

We use the clusterProfiler software package in R [21] for Gene Ontology (GO) enrichment and KEGG analysis to obtain GO terms (biological processes, molecular function, and cellular component) and KEGG pathways. Significance was defined as P < 0.05.

### Establishment and verification of the prognostic prediction model

According to the coxph function of the survival package in R, combined with the methylation characteristics and prognostic information of the three CpGs, a Cox proportional hazard model was constructed. For this model, we use the formula as: risk score = 2.4139 × cg18312429 + 2.0075 × cg19826026 + 2.4836 × cg27562023. The ROC packet in R was used to establish a receiver operation characteristic (ROC) curve. To verify the reliability as well as stability of this model, 204 testing samples were analyzed using this prediction model.

### Generation and validation of a predictive nomogram

In this study, univariate and multivariate Cox regression analyses were performed to determine independent prognostic factors to construct the nomogram. All independent prognostic factors obtained by multivariate COX regression analysis were selected to construct a combined prognostic model to evaluate the probability of 1-year, 3-year, and 5-year OS in patients with BCA. To evaluate the consistency between the actual survival rate and the predicted survival rate, a calibration curve can be drawn in which the 45° line represents the best prediction and the coincidence represents the better predicted value of the model.

## Results

### Identification prognostic methylation sites associated with OS

The prognostic molecular subsets were clustered based on a DNA methylation map of BCA samples in the TCGA database. Successfully, a total of 21,122 CpG sites were screened for methylation based on 203 experimental samples in the training group. The univariate Cox proportional hazard regression model was applied to each CpG site in the training group. Here, 1096 important CpG sites that might affect the survival of patients (P < 0.001) were identified. Accordingly, 1096 significant CpG sites were taken into the multivariate Cox proportional hazard regression model to study independent prognostic factors (P < 0.001). Finally, 402 meaningful CpG sites were obtained for further prognostic subgroup analysis (Additional file 1: Table S1).

### Identification of different DNA methylation prognostic subgroups through consensus clustering and prognostic analyses between clusters

**Fig. 1:**
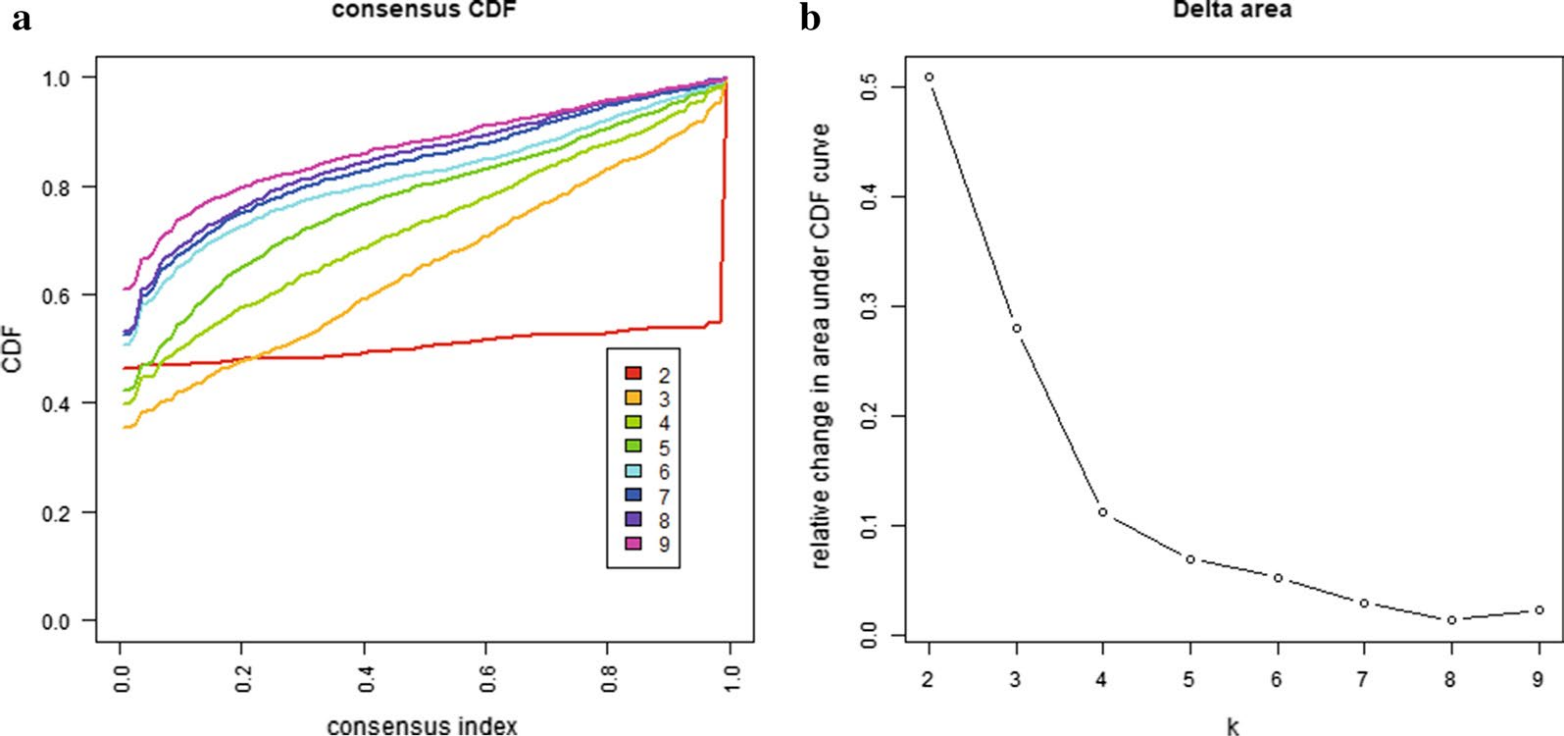
Consensus clustering of distinct bladder cancer DNA methylation prognostic subgroups. **a** Cumulative distribution function (CDF) curve. **b** CDF delta area curve. The delta area curve of consensus clustering shows the relative change in the area under the CDF curve for each category number k compared to that of k-1. The horizontal axis represents the category number k, and the vertical axis represents the relative change in the area under the CDF curve

**Fig. 2:**
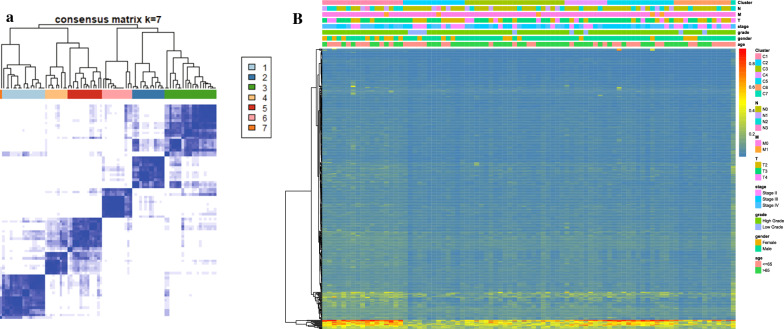
Cluster analysis of seven molecular subtypes by DNA methylation classification with the corresponding heat maps. **a** Color-coded heat map corresponding to the consensus matrix of seven molecular subtypes, obtained by applying consensus clustering. **b** The function of the heatmap annotated based on DNA methylation classification, TNM staging, clinicopathological staging, and histological type

**Fig. 3:**
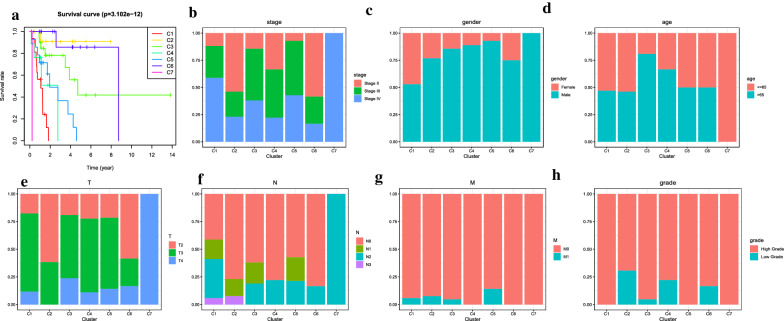
Comparison of prognosis, TNM stage, sex, age, and grade among the DNA methylation clusters. **a** Survival curves for each DNA methylation subtype. **b** Proportion of each clinical stage among the seven clusters. **c** Proportion of both sexes among the seven clusters. **d** Proportions of different ages among the seven clusters. **e** Proportions of different T stage degrees among the seven clusters. **f** Proportions of different N stage degrees among the seven clusters. **g** Proportions of different M stage degrees among the seven clusters. **h** Proportions of different pathological grades among the seven clusters

The consensus clustering of 402 potential prognostic methylation sites was performed to identify different DNA methylation molecular subsets and for further prognostic analyses. The optimal class number was obtained by calculating the average cluster consistency and intercluster coefficient of variation of each class number. As displayed by the CDF curve, when the clustering number is 7, the clustering result is relatively stable (Fig. 1a). Further observation of the CDF delta area curve shows that the area under the CDF curve starts to remain stable after seven categories, suggesting that when the cluster is selected as 7, the cluster has stable clustering results, and the seven categories were also considered the ideal number of categories for further analysis (Fig. 1b). The consensus matrix, as shown in Fig. 2a, illustrates the consensus of K = 7 and shows the superlative seven-block structure. Moreover, the heatmaps in accordance with 402 CpGs were generated via the heatmap function based on DNA methylation classification with T category, N category, M category, grade, stage, sex and age (Fig. 2b). The Kaplan–Meier diagram illustrated that the prognosis of BCA, defined by consensus clustering based on methylation, had significant differences among the seven clusters (P < 0.05; Fig. 3a). According to our results, the prognosis of Clusters 2 and 6 were the best, whereas that of Cluster 7 was the worst. Figure 3b–h indicates the intracluster proportions for the seven clusters on the basis of T category, N category, M category, grade, stage, sex, and age. The association trends between features and specific clusters were as follows: Clusters 1 and 7 were in advanced stage; Clusters 2 had lower T grade; Clusters 7 had higher N grade, younger age, and more male; Cluster 5 was in higher M grade; Clusters 1, 5, and 7 had higher grade.

### Differential feature recognition based on DNA methylation clustering and screening of cluster-specific methylation sites

A total of 557 promoter genes were identified according to the genome annotation of 402 meaningful CpG sites; the detailed gene list is given in Additional file 2: Table S2. The most significant GO terms for biological process, cellular component, and molecular function are shown in Additional file 3: Figure S1a–c. Next, we analyzed the functional enrichment of these 557 genes and recognized 23 significantly enriched pathways (P < 0.05; Additional file 3: Figure S1d). We observed that the three most significantly enriched pathways were peroxisome, base excision repair, and fatty acid biosynthesis pathways.

The expression of methylated genes found in the subgroup were also studied. Expression values were available for 465 of the 557 genes in the training group. We also generated a heatmap for specific annotated genes with methylation sites, as shown in Additional file 4: Figure S2, with different gene expression patterns among different subgroups.

**Fig. 4:**
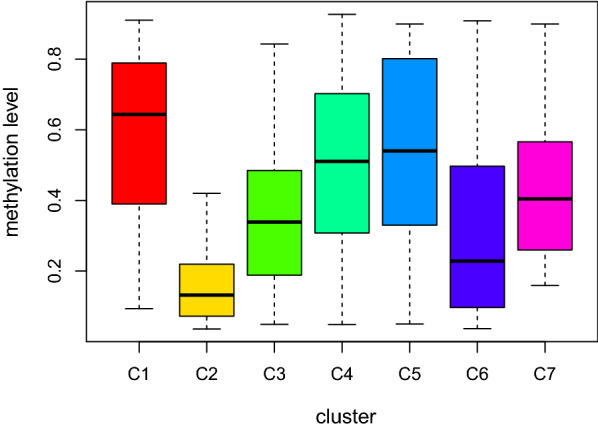
Box plot of CpG methylation levels in the seven clusters

The cluster-specific methylation sites were screened by using methylation sites as the feature of the clusters. The differences among seven clusters were analyzed for each methylation site. Eighteen cluster-specific methylation sites were identified and are shown in the heatmap in Additional file 5: Figure S3. The CpG sites enriched in each cluster are shown in Additional file 6: Table S3. Clusters 1, 2, and 6 had more specific sites, Cluster 1 had the highest level of methylation among all clusters, and Cluster 2 had the lowest methylation level among all clusters (Fig. 4).

### Construction and validation of the prognosis prediction model

**Fig. 5:**
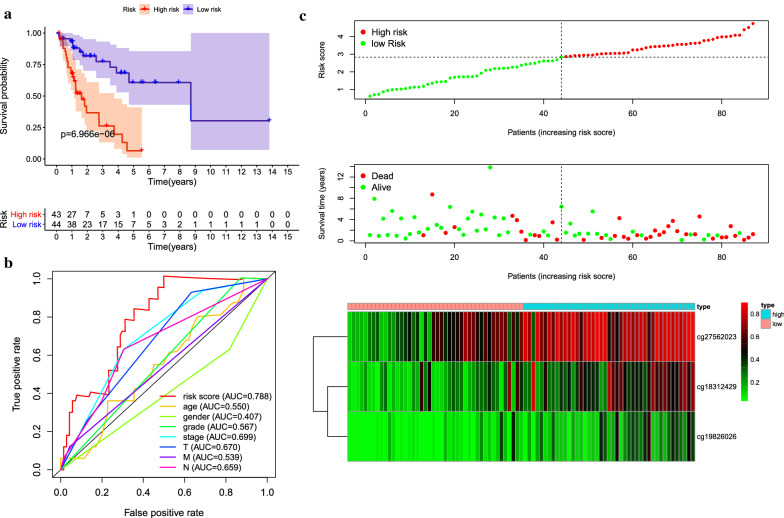
Construction of the prognostic prediction model for the training group. **a** Kaplan–Meier survival analysis of high- and low-risk groups in the training group. **b** Time-dependent receiver operation characteristic (ROC) of the indicated predictive factors in the training group. **c** Rank of risk score and distribution of groups, survival status of patients in different groups, and expression heatmap of the three CpGs sites included

Cluster 2 was found to contain a great number of samples that were associated with good prognosis and the highest number of specific methylation sites, which made it as seed cluster. Using the formula provided in the Methods section, we constructed a Cox proportional hazard model based on the methylation distribution of three specific sites combined with prognostic information. As shown in Fig. 5a, there was a significant difference in prognosis between the two groups. The results of ROC analysis using the risk score calculated for each sample are shown in Fig. 5b. The area under the curve (AUC) of the model was 0.788, which was higher than that of other predictive factors, indicates that the function of the model is effective. The samples were divided into high-risk group and low-risk group by median risk score which was used as cutoff value and then generate the risk curve (Fig. 5c).

**Fig. 6:**
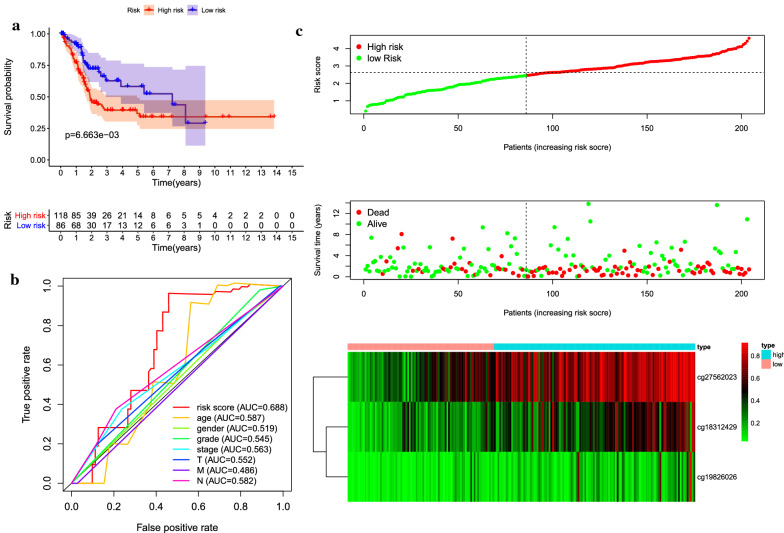
Construction of the prognostic prediction model for the testing group. **a** Kaplan–Meier survival analysis of high- and low-risk groups in the testing group. **b** Time-dependent receiver operation characteristic (ROC) of the indicated predictive factors in the testing group. **c** Rank of risk score and distribution of groups, survival status of patients in different groups, and expression heatmap of the three CpGs sites included

Next, the prognostic model was used to predict the outcomes of patients in the test dataset. The methylation level curves of three CpGs were obtained to test the dataset samples, and the prognostic model was used to calculate the risk score. The test dataset samples were then drawn into high-risk group and low-risk group. It is worth noting that there was a significant difference in prognosis between the two groups (P = 0.00666). It was consistent with the results gained from the training data set, indicating the accuracy of prediction and stability of the model (Fig. 6a–c).

### Clinical application of a nomogram

**Fig. 7:**
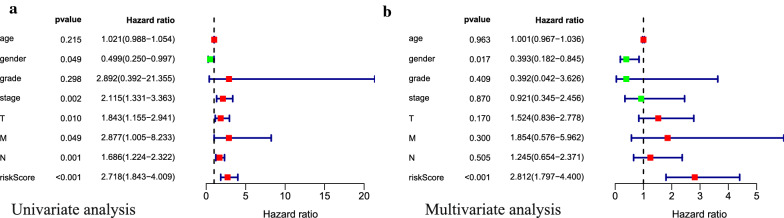
Univariate and multivariate COX regression analyses of bladder cancer cohort. Results showed that sex and the prognostic model were independent factors for predicting the overall survival rate of bladder cancer patients

**Fig. 8:**
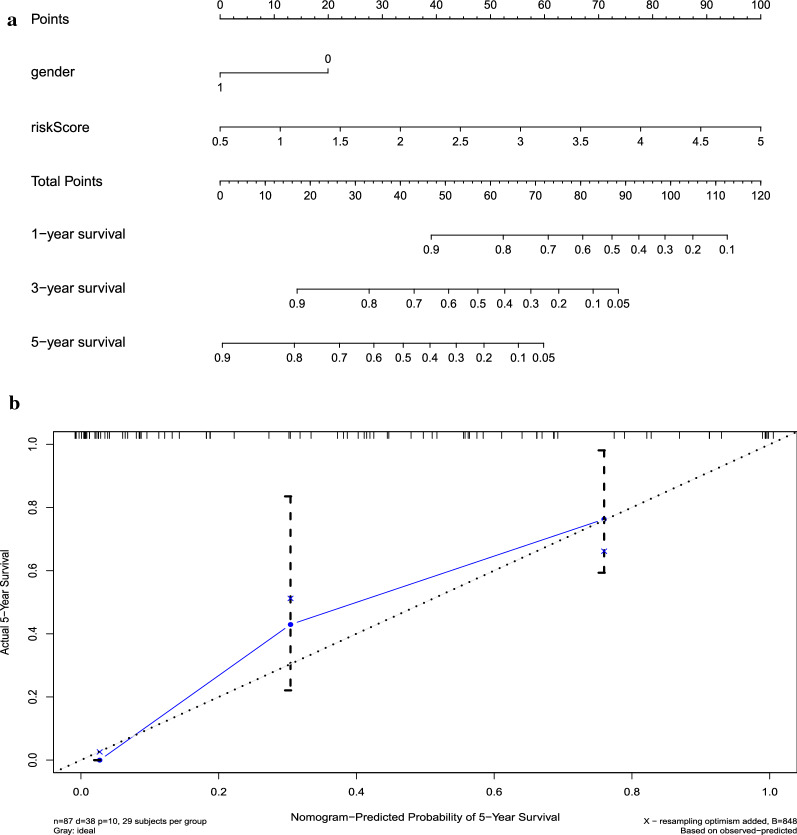
Nomogram and calibration plots **a** Nomogram to predict the probability of overall survival (OS) in patients with bladder cancer. **b** Calibration plots for the nomogram at 5 years

Through univariate and multivariate Cox regression analyses (Fig. 7a, b), both our prognostic model and sex were identified as potentially independent predictors, based on which nomogram was constructed (Fig. 8a). The C index was 0.77, and the correction chart showed that the actual survival rate of 5 years was in good agreement with the predicted survival rate (Fig. 8b). This indicates that the nomogram has high potential for clinical application.

## Discussion

The classical view of cancer evolution is that a series of genetic changes promotes the transition from the early precancerous stage to invasive cancer and affects the incidence of metastatic diseases. During carcinogenesis, oncogenes can be activated to promote cell division or inhibit cell death. At the same time, tumor suppressor genes can be inactivated in a way that can promote abnormal cell proliferation. Therefore, both the functional gain of proto-oncogene mutations and loss-of-function mutations in tumor suppressor genes might cause cancer through uncontrolled cell growth and defective apoptosis [22]. DNA methylation involves the addition of a methyl group at the cytosine 5′ carbon position of CpG dinucleotides in the genome, which is an important element of epigenetic regulation of gene expression [23]. Since the 1990s, increasing number of studies have recognized that heritable changes regulated by epigenetics might also play a vital role in the evolution of all types of human cancer [24]. We now know that epigenetic changes occur through specific events, including early widespread loss of normal DNA methylation and an increased number of focal gains in gene promoters [11].

BCA is a malignant tumor of the bladder that originates from the transformation of transitional intraepithelial urothelial cells, and is therefore also known as urothelial carcinoma or transitional cell carcinoma. Although transitional cell carcinoma of the bladder ranks fourth among men [2], the mechanism of the occurrence and development of urothelial carcinoma is still not completely clear. The development of modern techniques for genome-wide DNA methylation detection enables a more in-depth analysis of BCA methylation. Wolff et al. confirmed that the majority of DNA methylation changes occured in the early stage of BCA which were conserved in carcinoma in situ, non-invasive tumors and invasive tumors, and were located on the CpG island [25]. Compared to the urothelium from a healthy bladder, the hypermethylation of *ZO2*, *MYOD*, and *CDH13* were also detected in the urothelium with a normal appearance in patients with BCA, suggesting that epigenetic ‘field defects’ might be one of the reasons for the loss of epithelial integrity. Changes in DNA methylation comprise an early driver of cancer, and epigenetic changes involving DNA methylation might result in subsequent genome changes, which create a permissible environment for the onset and recurrence of BCA [25, 26]. In an interesting study, the gene methylation pattern of secondary bladder recurrence of primary upper urinary tract cancer was tested and it was confirmed that the methylation rate of some genes increased with the increase in the number of recurrences, which might be a predictor of postoperative recurrence [27]. Further, the methylation status of *GP5* and *ZSCAN12* can effectively be used to distinguish between high-grade and low-grade BCA [28]. At the same time, the methylation level of genes can effectively identify the degree of invasion of bladder tumors [25]. It is worth noting that based on TCGA data, the level of methylation and expression of *SOWAHC* is associated with prognosis [29]. *HOXA9* promoter methylation has also been shown to be associated with an increase in recurrence and progression in NMIBC. Importantly, it was also proved to be related to cisplatin resistance in BCA cells [30, 31]. In distinguishing whether patients with BCA have lymph node metastasis, a three-gene methylation panel was shown to predict the progression of metastasis and allow patients to benefit from lymphadenectomy and neoadjuvant chemotherapy [32].

In the enrichment analysis, the three most significant pathways identified were peroxisome, base excision repair, and fatty acid biosynthesis pathways. At present, it is believed that the peroxisome pathway mainly inhibits tumor proliferation, metastasis, and invasion by activating the expression of *PTEN, c*-*myc*, and *p27* [33, 34]. The peroxisome pathway has also been proved to be closely related to the occurrence and development of bladder cancer, and its expression is significantly increased in bladder cancer; moreover, its expression is higher in high-grade and invasive bladder cancer than in low-grade and superficial tumors [35]. Base excision repair plays a key role in maintaining genome stability, integrity, and preventing carcinogenesis, and DNA destruction may lead to gene rearrangement, translocation, amplification, and deletion [36]. Hence, defects in these genes may lead to higher susceptibility to multiple cancers [37]. Notably, a study involving 801 bladder cancer patients and 801 matched controls found that genetic variations in the BER pathway gene regulate the risk of bladder cancer [38]. Enrichment analysis showed that the genes related to methylation sites were highly related to the biological metabolism of fatty acids. Previous studies have shown that fatty acid metabolism plays an important role in maintaining the growth, migration, and invasion of bladder tumor cells [39]. Related metabonomic analysis and studies show that regulating fatty acid metabolism has a broad application prospect in the treatment and diagnosis of BCA [40, 41].

We used CpG sites to identify seven different prognostic subtypes of BCA, which could predict survival of the disease, as well as the TNM classification, grade, stage, and age distribution of prognosis among the seven molecular subtypes. Thus, this classification method results in molecular stratification that is suitable for a single tumor, which has an important impact on treatment decisions and accurate diagnoses. According to the seven molecular subtypes, if the important CpG sites were classified into category 7, the patients were found to have poor staging (more inclined to Stage IV), higher grade, a higher probability of lymph node metastasis, and poor prognosis. This is of great significance for early intervention and to actively encourage patients to receive treatment. Therefore, the radical resection of BCA and early lymph node dissection can be performed more actively. If the sequence of the CpG sites are classified into category 5, the risk of tumor metastasis is higher. Therefore, radiotherapy and chemotherapy should be actively performed to reduce the incidence of metastasis. However, when the sequence of the CpGs are classified as category 2, the tumor has lower invasiveness and the patient has a higher 5-year survival rate, indicating a better prognosis. This classification can motivate doctors to reconsider the individualized treatment of patients, conduct close clinical follow-up, and minimize overtreatment, which would help to reduce pain for the patients. In summary, our study of these seven subtypes at the DNA methylation molecular level indicated that this system could be used to more accurately classify BCA and guide clinicians in the diagnosis, treatment, and prognosis of different epigenetic subtypes.

With the accumulation of knowledges on the biology, function, and regulatory mechanisms of epigenetic modifications in cancer, and considering the limited efficacy of chemotherapy and immunotherapy, clinical success has ushered in an era of epigenetic therapy aimed at reactivating genes that are improperly silenced during carcinogenesis [42]. One of the first identified histone lysine methyltransferase inhibitors specific for G9a (EHMT2) is BIX-01294, which was considered to inhibit the proliferation of BCA cell lines [43]. In recent years, CM272 has been considered a novel double inhibitor of G9a/DNMT1, and it has significant antitumor effects on BCA in vivo and in vitro [44]. Demethylation drugs can inhibit the proliferation, migration, and invasion of BCA cells and have been shown to enhance the sensitivity of cisplatin-resistant cells [31, 45]. Using these concepts, DNA demethylation reagents are expected to be used in new therapeutic applications and might play an important role in cancer treatment. Therefore, the cancer epigenome as a target for cancer treatment still represents a vital opportunity for clinical exploitation, and exciting progress is expected in the next few years.

## Conclusions

We identified the methylation sites related to prognosis in patients with BCA and constructed a predictive model for these patients. The molecular subtypes based on methylation sites were found to be closely related to clinicopathology and could better predict the progression of BCA. Finally, this new targeting strategies will bring new horizons for BCA management and detection.

## Supplementary information

**Additional file 1: Table S1.** CpG sites were significant in multivariate Cox regression analyses.

**Additional file 2: Table S2.** 557 corresponding promotor genes.

**Additional file 3: Figure** S**1.** Enriched GO terms and KEGG pathways. **a** GO terms for biological process. **b** GO terms for cellular component. **c** GO terms for molecular function. **d** KEGG pathways.

**Additional file 4: Figure S2.** Heatmap of annotated genes associated with the 456 CpGs.

**Additional file 5: Figure S3**. Specific methylation of CpGs for each DNA methylation cluster. Specific CpGs are shown for each DNA methylation prognostic subtype. Red and blue represent hyper- and hypomethylated CpGs, respectively.

**Additional file 6: Table S3.** The distribution of samples and cluster-specific CpG sites based on 7 prognosis subgroups in the training groups

## Data Availability

For this study, Samples from the TCGA database containing 437 BCA methylation data are downloaded from UCSC Cancer Browser (http://xena.ucsc.edu/,2020-02-23). RNA-sequencing data from 433 BCA samples were downloaded from TCGA (https://cancergenome.nih.gov/, 2020-02-23).

## Abbreviations

AUC: Area under the curve
BCA: Bladder cancer
CpGs: CpGs
MIBC: Muscular invasive bladder cancer
NMIBC: Non-muscular invasive bladder cancer
ROC: Receiver operation characteristic
