# Supplementary material for: DNA methylation-based classification and identification of bladder cancer prognosis-associated subgroups
Source: Cancer Cell Int. 2020 Jun 17;20:255. doi: 10.1186/s12935-020-01345-1 (PMC7302382; doi:10.1186/s12935-020-01345-1)
Supplement: Supplementary file 1 — Additional file 1: Table S1. CpG sites were significant in multivariate Cox regression analyses. [file 12935_2020_1345_MOESM1_ESM.docx]

**Additional file 1: Table S1 CpG sites were significant in multivariate Cox regression analyses**

| id | HR | HR.95L | HR.95H | pvalue |
| --- | --- | --- | --- | --- |
| cg02878987 | 2.07477E+19 | 1.69541E+12 | 2.54E+26 | 9.21E-08 |
| cg04108502 | 1.53E+22 | 1.86173E+13 | 1.26E+31 | 1.08E-06 |
| cg11802692 | 2.46874E+19 | 2.05254E+11 | 2.97E+27 | 2.55E-06 |
| cg07285838 | 2.94E+34 | 8.31041E+19 | 1.04E+49 | 3.43E-06 |
| cg00274399 | 5.01E+25 | 6.42061E+14 | 3.91E+36 | 3.76E-06 |
| cg08478189 | 4.90E+30 | 4.76516E+17 | 5.03E+43 | 3.79E-06 |
| cg01618304 | 3.95126E+18 | 45892313932 | 3.40E+26 | 4.36E-06 |
| cg05603623 | 2.71527E+11 | 3554389.162 | 2.07425E+16 | 4.45E-06 |
| cg04315264 | 1.5838E+12 | 9607765.697 | 2.61082E+17 | 4.58E-06 |
| cg20109151 | 4.94E+22 | 6.97383E+12 | 3.50E+32 | 6.32E-06 |
| cg22153481 | 3.80E+38 | 6.53E+21 | 2.21E+55 | 6.47E-06 |
| cg03756121 | 3.15156E+15 | 515235920.7 | 1.93E+22 | 7.60E-06 |
| cg09486567 | 7.22E+23 | 2.55867E+13 | 2.04E+34 | 7.66E-06 |
| cg22734315 | 1.47507E+15 | 323024949.6 | 6.74E+21 | 8.03E-06 |
| cg04883450 | 1.05238E+16 | 849933413.7 | 1.30E+23 | 9.54E-06 |
| cg14817848 | 2993055975 | 189477.2774 | 4.72795E+13 | 9.71E-06 |
| cg17709676 | 1.74932E+19 | 46841454000 | 6.53E+27 | 1.08E-05 |
| cg20095338 | 3408068.915 | 4174.245381 | 2782522988 | 1.10E-05 |
| cg15271594 | 1.04E+21 | 4.40756E+11 | 2.46E+30 | 1.11E-05 |
| cg27321931 | 3.57612E+14 | 113548222.1 | 1.13E+21 | 1.14E-05 |
| cg06983551 | 350198.632 | 1131.154868 | 108419355.6 | 1.28E-05 |
| cg23489497 | 4.17106E+11 | 2503801.337 | 6.94852E+16 | 1.29E-05 |
| cg12867068 | 2.27E+25 | 8.8075E+13 | 5.84E+36 | 1.33E-05 |
| cg14086033 | 5.63078E+14 | 127647212.3 | 2.48E+21 | 1.36E-05 |
| cg04565661 | 6.73653E+17 | 5927559165 | 7.66E+25 | 1.44E-05 |
| cg21306218 | 1.28E+43 | 2.70E+23 | 6.11E+62 | 1.76E-05 |
| cg08596544 | 5.82785E+19 | 53613304250 | 6.33E+28 | 1.81E-05 |
| cg20803370 | 1.42E+26 | 1.56002E+14 | 1.30E+38 | 1.82E-05 |
| cg12477012 | 6.18338E+18 | 15156654615 | 2.52E+27 | 1.89E-05 |
| cg19638349 | 9.90E+26 | 4.02547E+14 | 2.44E+39 | 1.95E-05 |
| cg01183669 | 7.38373E+19 | 54062111346 | 1.01E+29 | 2.02E-05 |
| cg01733783 | 1.43755E+16 | 525563663.5 | 3.93E+23 | 2.06E-05 |
| cg25887294 | 1.61698E+12 | 3850997.784 | 6.7895E+17 | 2.09E-05 |
| cg22774127 | 1.01942E+18 | 4556928001 | 2.28E+26 | 2.37E-05 |
| cg07962043 | 5.15812E+15 | 254983778.8 | 1.04E+23 | 2.50E-05 |
| cg13221588 | 18137696039 | 284711.7058 | 1.15547E+15 | 2.85E-05 |
| cg11628034 | 1.03E+22 | 4.98528E+11 | 2.15E+32 | 2.89E-05 |
| cg09593402 | 5.02219E+17 | 2507004396 | 1.01E+26 | 2.93E-05 |
| cg22892237 | 1.77108E+19 | 15657404448 | 2.00E+28 | 3.09E-05 |
| cg05501276 | 1.41549E+14 | 29409345.94 | 6.81E+20 | 3.32E-05 |
| cg10394385 | 2.80221E+19 | 17914798524 | 4.38E+28 | 3.39E-05 |
| cg21549639 | 6.88502E+12 | 5718868.895 | 8.28896E+18 | 3.50E-05 |
| cg12085660 | 2.64829E+19 | 15946420702 | 4.40E+28 | 3.65E-05 |
| cg09155852 | 1.58E+20 | 34896569892 | 7.19E+29 | 4.14E-05 |
| cg08759274 | 1.08811E+12 | 1884219.329 | 6.28368E+17 | 4.23E-05 |
| cg13285447 | 4.31E+31 | 3.07896E+16 | 6.03E+46 | 4.25E-05 |
| cg00317680 | 1.02094E+13 | 5924930.471 | 1.75921E+19 | 4.34E-05 |
| cg26942392 | 273891.023 | 674.3891063 | 111235919.7 | 4.40E-05 |
| cg26964415 | 1.42756E+16 | 240460178.8 | 8.48E+23 | 4.64E-05 |
| cg08358671 | 1970104.972 | 1836.405197 | 2113538780 | 4.68E-05 |
| cg13014406 | 2.63941E+15 | 97073454.18 | 7.18E+22 | 4.79E-05 |
| cg19110355 | 3.21E+26 | 5.25612E+13 | 1.97E+39 | 4.84E-05 |
| cg08552121 | 5.85E+20 | 53742140812 | 6.37E+30 | 5.01E-05 |
| cg12588476 | 5.46112E+16 | 439129200.4 | 6.79E+24 | 5.07E-05 |
| cg11039072 | 7.40E+23 | 2.10261E+12 | 2.61E+35 | 5.09E-05 |
| cg25438415 | 232.0362956 | 16.60787636 | 3241.886038 | 5.16E-05 |
| cg14203758 | 9.41707E+18 | 6088489943 | 1.46E+28 | 5.19E-05 |
| cg04785461 | 2.18024E+18 | 2776953997 | 1.71E+27 | 5.33E-05 |
| cg21459921 | 1.36E+24 | 2.33813E+12 | 7.86E+35 | 5.80E-05 |
| cg01195127 | 1.15441E+18 | 1727341931 | 7.72E+26 | 6.03E-05 |
| cg03954150 | 7.99E+29 | 1.82051E+15 | 3.50E+44 | 6.26E-05 |
| cg15563057 | 1.46762E+12 | 1597159.047 | 1.34859E+18 | 6.37E-05 |
| cg20273774 | 1.33505E+11 | 469135.2164 | 3.79922E+16 | 6.39E-05 |
| cg04743063 | 89885774642 | 381969.5818 | 2.11521E+16 | 6.42E-05 |
| cg25622628 | 853055.5789 | 1049.457472 | 693409538 | 6.48E-05 |
| cg10220156 | 2.47E+30 | 2.92853E+15 | 2.08E+45 | 6.58E-05 |
| cg15109571 | 9.24E+39 | 2.17E+20 | 3.93E+59 | 6.59E-05 |
| cg03079549 | 4.69E+22 | 3.36247E+11 | 6.54E+33 | 6.69E-05 |
| cg12830829 | 3820212.677 | 2217.732262 | 6580607205 | 6.71E-05 |
| cg27562023 | 94.09963969 | 10.073263 | 879.0341516 | 6.72E-05 |
| cg21028326 | 4239549571 | 77455.82901 | 2.32052E+14 | 6.83E-05 |
| cg15654964 | 1.31267E+14 | 14577751.22 | 1.18E+21 | 6.92E-05 |
| cg17846100 | 7.56333E+19 | 12082041491 | 4.73E+29 | 6.98E-05 |
| cg21878918 | 155324961.3 | 14172.13463 | 1.70234E+12 | 7.06E-05 |
| cg01932459 | 124268442 | 12442.19038 | 1.24115E+12 | 7.29E-05 |
| cg04273556 | 6.47165E+11 | 933587.3323 | 4.48616E+17 | 7.39E-05 |
| cg15584630 | 5.76E+48 | 4.04E+24 | 8.21E+72 | 7.60E-05 |
| cg03366677 | 6.93E+69 | 1.65E+35 | 2.91E+104 | 7.70E-05 |
| cg09576882 | 6.36375E+15 | 92152935.9 | 4.39E+23 | 7.77E-05 |
| cg27281008 | 7.68563E+16 | 309450599.1 | 1.91E+25 | 8.07E-05 |
| cg06310844 | 102065.2741 | 328.3422547 | 31727016.65 | 8.20E-05 |
| cg04982748 | 2.23011E+15 | 50732379.02 | 9.80E+22 | 8.29E-05 |
| cg12104707 | 277.0153594 | 16.70564027 | 4593.509025 | 8.67E-05 |
| cg22907065 | 7.80E+66 | 3.02E+33 | 2.02E+100 | 8.71E-05 |
| cg08207256 | 2657599244 | 51902.65562 | 1.36078E+14 | 8.77E-05 |
| cg21693780 | 2.32E+20 | 15017570306 | 3.57E+30 | 8.94E-05 |
| cg20760063 | 725471.706 | 843.683802 | 623822805.3 | 9.06E-05 |
| cg08440125 | 3.65E+26 | 1.78445E+13 | 7.46E+39 | 9.18E-05 |
| cg27072323 | 185676478.5 | 12920.60351 | 2.66828E+12 | 9.69E-05 |
| cg07289581 | 2.94E+25 | 4.46789E+12 | 1.94E+38 | 9.85E-05 |
| cg03989322 | 23533963294 | 141436.393 | 3.91588E+15 | 9.88E-05 |
| cg24914860 | 4.76E+25 | 5.60087E+12 | 4.04E+38 | 9.92E-05 |
| cg06659073 | 27143701156 | 151297.0354 | 4.86976E+15 | 9.93E-05 |
| cg13415434 | 4.79999E+14 | 19241238.7 | 1.20E+22 | 0.0001 |
| cg13650137 | 40272801.52 | 5931.769606 | 2.73426E+11 | 0.0001 |
| cg15210596 | 3.08E+35 | 3.96525E+17 | 2.39E+53 | 0.000101 |
| cg24148044 | 6.93E+21 | 67413005586 | 7.12E+32 | 0.000101 |
| cg25718402 | 3.38138E+12 | 1625104.429 | 7.03567E+18 | 0.000102 |
| cg06725760 | 5.85E+36 | 1.64848E+18 | 2.08E+55 | 0.000102 |
| cg08020381 | 3.81834E+16 | 162689617.4 | 8.96E+24 | 0.000103 |
| cg17660026 | 7555.977745 | 83.15441329 | 686587.7279 | 0.000104 |
| cg12927772 | 7.21E+26 | 1.97075E+13 | 2.63E+40 | 0.000104 |
| cg25786862 | 1.38112E+18 | 950582934.9 | 2.01E+27 | 0.000104 |
| cg22478328 | 3.53381E+19 | 4716551916 | 2.65E+29 | 0.000104 |
| cg07777378 | 3.53E+20 | 14499228676 | 8.58E+30 | 0.000106 |
| cg12583908 | 1.10E+31 | 2.17366E+15 | 5.60E+46 | 0.000107 |
| cg14085328 | 1.16482E+17 | 262484090.5 | 5.17E+25 | 0.00011 |
| cg00291877 | 1.62E+20 | 9220958887 | 2.85E+30 | 0.00011 |
| cg17466768 | 2892744.728 | 1532.837981 | 5459136689 | 0.000111 |
| cg14255089 | 2.07E+24 | 9.7231E+11 | 4.41E+36 | 0.000111 |
| cg25722142 | 1.96288E+13 | 3458828.483 | 1.11E+20 | 0.000115 |
| cg18580491 | 3.42E+73 | 1.05E+36 | 1.11E+111 | 0.000122 |
| cg15863254 | 4.72106E+16 | 145314477.9 | 1.53E+25 | 0.000123 |
| cg01087710 | 2953058663 | 42995.45224 | 2.02825E+14 | 0.000124 |
| cg00893987 | 8.88E+33 | 3.97929E+16 | 1.98E+51 | 0.000125 |
| cg20646490 | 484891022.1 | 17314.68565 | 1.35792E+13 | 0.000129 |
| cg07200897 | 4.82E+23 | 3.24095E+11 | 7.16E+35 | 0.000137 |
| cg17452277 | 5.78E+66 | 2.62E+32 | 1.27E+101 | 0.000139 |
| cg08293367 | 31402388.96 | 4349.233441 | 2.26732E+11 | 0.00014 |
| cg27268717 | 9.25536E+15 | 54892378.28 | 1.56E+24 | 0.000142 |
| cg18312429 | 36.42326765 | 5.707269232 | 232.4499463 | 0.000144 |
| cg18294881 | 1.56E+34 | 3.64698E+16 | 6.67E+51 | 0.000144 |
| cg00180012 | 3.39E+22 | 79754779246 | 1.44E+34 | 0.000146 |
| cg01998682 | 3.69E+58 | 2.07E+28 | 6.58E+88 | 0.000148 |
| cg15946545 | 68177695.09 | 6114.478206 | 7.60195E+11 | 0.000148 |
| cg03112782 | 7.18883E+19 | 3898807740 | 1.33E+30 | 0.00015 |
| cg03243895 | 3.36E+39 | 1.22E+19 | 9.23E+59 | 0.000151 |
| cg22017387 | 1.80352E+19 | 1938893742 | 1.68E+29 | 0.000153 |
| cg22552684 | 2.57292E+13 | 2930012.389 | 2.26E+20 | 0.000153 |
| cg14946299 | 42912841050 | 133595.1013 | 1.37843E+16 | 0.000154 |
| cg11054882 | 9.72E+23 | 3.6376E+11 | 2.60E+36 | 0.000155 |
| cg07569756 | 2.50295E+13 | 2852207.642 | 2.20E+20 | 0.000155 |
| cg18797053 | 1.98661E+17 | 214802107.2 | 1.84E+26 | 0.000156 |
| cg02470959 | 4.39097E+11 | 393544.7336 | 4.89922E+17 | 0.000161 |
| cg02372712 | 1.6902E+17 | 189625382.9 | 1.51E+26 | 0.000161 |
| cg01117627 | 55765073.89 | 5272.804908 | 5.8977E+11 | 0.000161 |
| cg22062239 | 1.72E+26 | 4.00569E+12 | 7.38E+39 | 0.000162 |
| cg16208491 | 1.02E+24 | 3.27417E+11 | 3.16E+36 | 0.000165 |
| cg23640903 | 9.47359E+13 | 4993881.874 | 1.80E+21 | 0.000167 |
| cg10215884 | 12599036256 | 69213.86554 | 2.29341E+15 | 0.000168 |
| cg22938407 | 2.95E+43 | 6.51E+20 | 1.34E+66 | 0.00017 |
| cg12017007 | 1.85E+21 | 14841752809 | 2.31E+32 | 0.000172 |
| cg00833393 | 1.99185E+17 | 187610534.7 | 2.11E+26 | 0.000172 |
| cg17054360 | 18657547140 | 80959.0784 | 4.29975E+15 | 0.000174 |
| cg02917972 | 3.20049E+14 | 8399970.08 | 1.22E+22 | 0.000177 |
| cg24003306 | 5.49E+53 | 4.34E+25 | 6.95E+81 | 0.000178 |
| cg01491540 | 6.98E+43 | 7.77E+20 | 6.27E+66 | 0.000181 |
| cg02951021 | 27804344.4 | 3520.841612 | 2.19573E+11 | 0.000181 |
| cg17140815 | 1.88519E+17 | 169251071.9 | 2.10E+26 | 0.000182 |
| cg09788239 | 21413711.22 | 3065.174352 | 1.49599E+11 | 0.000186 |
| cg00107632 | 1.71E+23 | 1.08464E+11 | 2.70E+35 | 0.000189 |
| cg15792688 | 13854.99258 | 92.6327479 | 2072278.15 | 0.00019 |
| cg04145477 | 5.01E+21 | 19088241906 | 1.31E+33 | 0.000196 |
| cg18342900 | 1.75042E+16 | 49530953.11 | 6.19E+24 | 0.000196 |
| cg24459023 | 9.48671E+14 | 12391070.33 | 7.26E+22 | 0.000197 |
| cg12806381 | 4.48011E+14 | 8569079.198 | 2.34E+22 | 0.000199 |
| cg11082575 | 1.50E+20 | 3505362489 | 6.42E+30 | 0.0002 |
| cg05978187 | 2.50E+63 | 9.12E+29 | 6.88E+96 | 0.000202 |
| cg20482071 | 4.09E+35 | 6.5387E+16 | 2.56E+54 | 0.000205 |
| cg04660698 | 1.98164E+11 | 215308.3783 | 1.82385E+17 | 0.000205 |
| cg22091798 | 6.98878E+16 | 89136840.15 | 5.48E+25 | 0.000206 |
| cg22906389 | 3.75E+20 | 5115785616 | 2.74E+31 | 0.000206 |
| cg01883521 | 3.6789E+19 | 1676314673 | 8.07E+29 | 0.000209 |
| cg18251360 | 5.97082E+11 | 355865.9695 | 1.0018E+18 | 0.000209 |
| cg10485472 | 2.17E+30 | 1.98522E+14 | 2.38E+46 | 0.00021 |
| cg06422693 | 13299761151 | 58570.78363 | 3.02E+15 | 0.000212 |
| cg12813922 | 4.22899E+18 | 566319679.6 | 3.16E+28 | 0.000218 |
| cg21300561 | 2.58146E+16 | 50714668.15 | 1.31E+25 | 0.00022 |
| cg25841091 | 2.97E+23 | 1.045E+11 | 8.42E+35 | 0.000221 |
| cg12719235 | 1.49597E+18 | 336491355.3 | 6.65E+27 | 0.000222 |
| cg27556566 | 1.05738E+16 | 32888950.67 | 3.40E+24 | 0.000223 |
| cg25026754 | 3.54E+21 | 12378817614 | 1.01E+33 | 0.000227 |
| cg01046174 | 2.39229E+14 | 5408483.222 | 1.06E+22 | 0.000228 |
| cg07684150 | 3.11E+30 | 1.88238E+14 | 5.15E+46 | 0.000229 |
| cg04626574 | 2.28098E+18 | 381881511.9 | 1.36E+28 | 0.000233 |
| cg02212737 | 1.57584E+15 | 12551167.75 | 1.98E+23 | 0.000235 |
| cg22499237 | 3.05E+19 | 1255207614 | 7.41E+29 | 0.000236 |
| cg03930357 | 5.81E+23 | 1.24285E+11 | 2.71E+36 | 0.000237 |
| cg09693433 | 2.67E+26 | 2.17411E+12 | 3.29E+40 | 0.000237 |
| cg14576681 | 1.01721E+16 | 29705312.88 | 3.48E+24 | 0.000237 |
| cg14408969 | 1.68192E+11 | 172043.4436 | 1.64428E+17 | 0.00024 |
| cg13145440 | 1.25234E+12 | 435351.8931 | 3.60252E+18 | 0.000242 |
| cg25159610 | 5.22926E+12 | 845493.7333 | 3.23423E+19 | 0.000242 |
| cg23970338 | 2314398075 | 23079.72374 | 2.32084E+14 | 0.000243 |
| cg00432979 | 1.05E+50 | 1.90E+23 | 5.81E+76 | 0.000246 |
| cg20592700 | 1699.172125 | 31.61099984 | 91334.84944 | 0.000253 |
| cg00171161 | 2083841.398 | 855.71932 | 5074555254 | 0.000255 |
| cg22632663 | 1.81126E+14 | 4127027.549 | 7.95E+21 | 0.000256 |
| cg24818434 | 1.52E+32 | 8.54048E+14 | 2.71E+49 | 0.000256 |
| cg26350691 | 1.17915E+12 | 397722.7942 | 3.49591E+18 | 0.000256 |
| cg09765256 | 2.23097E+17 | 111479990.2 | 4.46E+26 | 0.000257 |
| cg08711724 | 2.22191E+16 | 37717513.95 | 1.31E+25 | 0.000259 |
| cg00123035 | 1.26846E+16 | 28345184.12 | 5.68E+24 | 0.000264 |
| cg15021531 | 4.7564E+17 | 151635709.5 | 1.49E+27 | 0.000264 |
| cg09455096 | 9.22E+23 | 1.22774E+11 | 6.92E+36 | 0.000264 |
| cg20684973 | 122108.7588 | 224.476005 | 66423798.74 | 0.000268 |
| cg21048501 | 2.77E+25 | 5.47068E+11 | 1.41E+39 | 0.000274 |
| cg24906202 | 528716710.6 | 10414.20146 | 2.68423E+13 | 0.00028 |
| cg23644127 | 1.43725E+13 | 1126264.264 | 1.83E+20 | 0.000284 |
| cg01333619 | 2.43112E+13 | 1427798.5 | 4.14E+20 | 0.000285 |
| cg11296937 | 9033.190154 | 65.71807817 | 1241645.018 | 0.000288 |
| cg03424436 | 5.85717E+18 | 419175457.8 | 8.18E+28 | 0.000288 |
| cg06440646 | 4.64282E+16 | 44938717.29 | 4.80E+25 | 0.00029 |
| cg01998146 | 2.73E+20 | 2410216960 | 3.09E+31 | 0.000291 |
| cg03386722 | 8.25E+25 | 7.71645E+11 | 8.83E+39 | 0.000294 |
| cg14931645 | 2.24123E+19 | 731296996.9 | 6.87E+29 | 0.000298 |
| cg20755353 | 1.15041E+13 | 957375.7415 | 1.38E+20 | 0.000299 |
| cg00047469 | 1.49723E+13 | 1077472.53 | 2.08E+20 | 0.0003 |
| cg25372103 | 2.62552E+12 | 481835.2489 | 1.43064E+19 | 0.000302 |
| cg15676241 | 1.27104E+13 | 973962.4988 | 1.66E+20 | 0.000307 |
| cg05577173 | 17524261724 | 47944.53875 | 6.40531E+15 | 0.000307 |
| cg16050957 | 2.45107E+12 | 457993.9071 | 1.31175E+19 | 0.000307 |
| cg13243377 | 1.70E+21 | 4899580120 | 5.93E+32 | 0.000311 |
| cg15994781 | 7.47417E+15 | 17104223.1 | 3.27E+24 | 0.000317 |
| cg23013931 | 1.51697E+16 | 23613379.32 | 9.75E+24 | 0.000317 |
| cg24695828 | 1.81746E+11 | 133947.7156 | 2.46601E+17 | 0.00032 |
| cg23152667 | 12108.35411 | 72.29429568 | 2027991.805 | 0.00032 |
| cg05923103 | 3.40539E+13 | 1449774.584 | 8.00E+20 | 0.00032 |
| cg00746981 | 3.13852E+11 | 170043.3905 | 5.7928E+17 | 0.000323 |
| cg09147213 | 17460435582 | 45405.57677 | 6.7143E+15 | 0.000325 |
| cg19522332 | 3.20E+24 | 1.37625E+11 | 7.43E+37 | 0.000326 |
| cg18333824 | 3.47E+28 | 9.35939E+12 | 1.29E+44 | 0.000327 |
| cg15118204 | 5.11991E+12 | 594003.4233 | 4.41302E+19 | 0.000329 |
| cg09664442 | 3.52E+36 | 3.95763E+16 | 3.12E+56 | 0.00033 |
| cg27403822 | 3.80E+23 | 50601551632 | 2.85E+36 | 0.000331 |
| cg03367667 | 7.29438E+19 | 1020879330 | 5.21E+30 | 0.000335 |
| cg16248277 | 1669.960337 | 28.94138503 | 96359.15917 | 0.000335 |
| cg01366407 | 1.58669E+13 | 959957.0062 | 2.62E+20 | 0.000338 |
| cg19020694 | 1.33E+40 | 1.47283E+18 | 1.21E+62 | 0.000341 |
| cg11368509 | 2.27E+24 | 1.06524E+11 | 4.82E+37 | 0.000341 |
| cg24142014 | 6.75692E+12 | 632426.4927 | 7.21917E+19 | 0.000347 |
| cg17223189 | 119732137.4 | 4435.728128 | 3.23189E+12 | 0.000353 |
| cg21824902 | 700667719.6 | 9809.295869 | 5.0048E+13 | 0.000355 |
| cg07281688 | 13295605.53 | 1637.819328 | 1.07932E+11 | 0.000355 |
| cg27367554 | 1.60393E+19 | 454429710.1 | 5.66E+29 | 0.000359 |
| cg02501984 | 2.45506E+12 | 383322.3803 | 1.57239E+19 | 0.00036 |
| cg17593391 | 389481565.8 | 7399.853436 | 2.04999E+13 | 0.000362 |
| cg19026306 | 1.40789E+13 | 835770.4165 | 2.37E+20 | 0.000362 |
| cg18349258 | 5.74279E+12 | 553234.375 | 5.96124E+19 | 0.000365 |
| cg21037570 | 1.44713E+16 | 18759918.24 | 1.12E+25 | 0.000365 |
| cg16108132 | 3.75274E+17 | 79778495.76 | 1.77E+27 | 0.000369 |
| cg23610841 | 9.88056E+19 | 975440960.2 | 1.00E+31 | 0.00037 |
| cg07401901 | 1.67E+20 | 1209843704 | 2.29E+31 | 0.000374 |
| cg02917381 | 2.11051E+19 | 477391794.6 | 9.33E+29 | 0.000374 |
| cg17150465 | 6.08E+20 | 2157810828 | 1.71E+32 | 0.000374 |
| cg11787828 | 1790924.289 | 630.7094227 | 5085400174 | 0.000387 |
| cg08265265 | 3.91E+49 | 1.60E+22 | 9.58E+76 | 0.000387 |
| cg11226328 | 631808711.7 | 8702.191981 | 4.58715E+13 | 0.000388 |
| cg00177013 | 9.26408E+18 | 309108184.1 | 2.78E+29 | 0.000388 |
| cg18424538 | 1.10E+63 | 1.60E+28 | 7.62E+97 | 0.00039 |
| cg15740366 | 3.57269E+14 | 3233288.405 | 3.95E+22 | 0.000391 |
| cg11475332 | 1.30E+37 | 3.75625E+16 | 4.50E+57 | 0.000398 |
| cg16109297 | 146517.7712 | 202.160311 | 106190266.4 | 0.0004 |
| cg26477793 | 2197865732 | 14741.19875 | 3.27695E+14 | 0.000401 |
| cg13846682 | 68135200.76 | 3075.367661 | 1.50954E+12 | 0.000411 |
| cg08793459 | 7161.53348 | 52.03494906 | 985636.8211 | 0.000411 |
| cg21144587 | 8293.912034 | 55.45662356 | 1240410.476 | 0.000413 |
| cg12582008 | 15.72827843 | 3.408019168 | 72.5872509 | 0.000413 |
| cg23213688 | 336835660.3 | 6189.046881 | 1.83321E+13 | 0.000417 |
| cg11061603 | 2.03E+25 | 1.71252E+11 | 2.41E+39 | 0.000424 |
| cg18337803 | 4.48E+50 | 3.03E+22 | 6.64E+78 | 0.000425 |
| cg16864895 | 3.92206E+17 | 63249490.34 | 2.43E+27 | 0.000429 |
| cg27007358 | 23588323.52 | 1848.425531 | 3.01018E+11 | 0.000433 |
| cg24490859 | 9.96E+23 | 42652165776 | 2.33E+37 | 0.000434 |
| cg03802191 | 152.7951331 | 9.238442908 | 2527.087403 | 0.000443 |
| cg20030671 | 2.14E+22 | 7268433252 | 6.28E+34 | 0.000448 |
| cg07125009 | 4.79E+28 | 4.51641E+12 | 5.08E+44 | 0.000452 |
| cg17718515 | 24530297118 | 38153.10635 | 1.57716E+16 | 0.000455 |
| cg04802801 | 7.03796E+17 | 74116915.81 | 6.68E+27 | 0.000455 |
| cg12477119 | 165978521.8 | 4206.657544 | 6.54887E+12 | 0.000456 |
| cg18652121 | 2.7881E+11 | 110755.9481 | 7.01857E+17 | 0.000457 |
| cg11880892 | 1.62507E+17 | 37796581.27 | 6.99E+26 | 0.000462 |
| cg11646757 | 3.10301E+12 | 311449.1323 | 3.09157E+19 | 0.000468 |
| cg00899086 | 28414797199 | 39080.78742 | 2.06598E+16 | 0.000473 |
| cg06894812 | 5.82E+34 | 1.85343E+15 | 1.83E+54 | 0.000474 |
| cg21939482 | 9.13665E+15 | 10174622.17 | 8.20E+24 | 0.000476 |
| cg24319545 | 6.13E+20 | 1337047046 | 2.81E+32 | 0.000476 |
| cg25969673 | 4.0685E+16 | 19200576.72 | 8.62E+25 | 0.000482 |
| cg20149998 | 3.14E+20 | 958653863.2 | 1.03E+32 | 0.000486 |
| cg05889321 | 21373831935 | 33407.77519 | 1.36747E+16 | 0.000488 |
| cg21664828 | 3.31089E+15 | 6167718.407 | 1.78E+24 | 0.000493 |
| cg03801144 | 3.88E+21 | 2783876363 | 5.42E+33 | 0.000494 |
| cg18380975 | 198012901.8 | 4238.429129 | 9.25086E+12 | 0.000497 |
| cg26703664 | 7.39E+30 | 3.10946E+13 | 1.76E+48 | 0.000498 |
| cg04875709 | 5.84E+67 | 4.10E+29 | 8.33E+105 | 0.000499 |
| cg14034870 | 107.2295654 | 7.706544206 | 1492.002043 | 0.000501 |
| cg21097640 | 6.40626E+11 | 140877.8792 | 2.91317E+18 | 0.000509 |
| cg12018696 | 2.08E+22 | 5222308876 | 8.29E+34 | 0.000517 |
| cg03328804 | 1.54798E+11 | 73439.34115 | 3.26287E+17 | 0.000524 |
| cg21612046 | 472.5607077 | 14.53005692 | 15369.08105 | 0.000527 |
| cg18061485 | 3.01324E+18 | 105322281.9 | 8.62E+28 | 0.000533 |
| cg11074746 | 2.67824E+19 | 262778916.6 | 2.73E+30 | 0.000542 |
| cg20314660 | 8.49E+51 | 3.12E+22 | 2.31E+81 | 0.000545 |
| cg03070588 | 2.53E+21 | 1854447744 | 3.44E+33 | 0.000546 |
| cg18119609 | 1.93868E+19 | 224102080.6 | 1.68E+30 | 0.000547 |
| cg19451466 | 2.83203E+13 | 663132.9329 | 1.21E+21 | 0.00055 |
| cg17460846 | 2.60E+20 | 667864697.3 | 1.01E+32 | 0.000556 |
| cg02235880 | 1171972675 | 8202.814204 | 1.67445E+14 | 0.000565 |
| cg01263716 | 1.28E+87 | 3.77E+37 | 4.34E+136 | 0.000567 |
| cg15824968 | 2.96414E+17 | 34317795.64 | 2.56E+27 | 0.000568 |
| cg09469394 | 1284310.933 | 430.3825323 | 3832531407 | 0.00057 |
| cg11468462 | 5.30E+43 | 6.97304E+18 | 4.03E+68 | 0.000572 |
| cg26069252 | 7.65941E+15 | 6917910.626 | 8.48E+24 | 0.000577 |
| cg05610148 | 4.53E+22 | 5675464129 | 3.62E+35 | 0.000578 |
| cg16900022 | 5.77217E+15 | 6077895.12 | 5.48E+24 | 0.00058 |
| cg24650353 | 1.76E+28 | 1.42072E+12 | 2.18E+44 | 0.000582 |
| cg24341944 | 2358324.912 | 547.5967657 | 10156554488 | 0.000588 |
| cg18296405 | 8.41E+39 | 1.34061E+17 | 5.28E+62 | 0.000598 |
| cg17158059 | 1.2821E+19 | 157220357.2 | 1.05E+30 | 0.000599 |
| cg19864851 | 4.30938E+16 | 13622240.34 | 1.36E+26 | 0.0006 |
| cg22377389 | 523.9729084 | 14.64150694 | 18751.32184 | 0.000603 |
| cg08661227 | 8.60E+29 | 6.58783E+12 | 1.12E+47 | 0.000608 |
| cg18805835 | 2.04776E+13 | 500756.8444 | 8.37E+20 | 0.000609 |
| cg25430696 | 3.89043E+16 | 12517769.73 | 1.21E+26 | 0.000614 |
| cg22973038 | 2.00E+25 | 65938424086 | 6.06E+39 | 0.000617 |
| cg08626943 | 9383872907 | 18341.82322 | 4.80089E+15 | 0.000618 |
| cg03538345 | 15642.42985 | 61.79329285 | 3959743.856 | 0.000625 |
| cg18512092 | 9.71E+25 | 1.20679E+11 | 7.81E+40 | 0.000633 |
| cg17441778 | 15760431808 | 22266.8844 | 1.11552E+16 | 0.000634 |
| cg02943497 | 9.32922E+13 | 900651.1162 | 9.66E+21 | 0.000635 |
| cg25834568 | 2.42971E+14 | 1314028.087 | 4.49E+22 | 0.000648 |
| cg25682936 | 1.69E+22 | 2820356243 | 1.01E+35 | 0.000651 |
| cg24654547 | 387931.3874 | 236.9989695 | 634984876.3 | 0.000654 |
| cg11233228 | 5.32229E+16 | 12658983.05 | 2.24E+26 | 0.000658 |
| cg08568987 | 3.4647E+15 | 3913656.619 | 3.07E+24 | 0.000664 |
| cg11266874 | 6.16613E+15 | 4859372.767 | 7.82E+24 | 0.000675 |
| cg20951821 | 4225342.768 | 638.4195085 | 27965187883 | 0.000677 |
| cg27467734 | 9.38505E+12 | 307562.0624 | 2.86E+20 | 0.000681 |
| cg09163442 | 6.0243E+14 | 1783279.202 | 2.04E+23 | 0.000682 |
| cg00426056 | 3.3619E+13 | 518118.9501 | 2.18E+21 | 0.00069 |
| cg27316224 | 1.53E+33 | 1.03768E+14 | 2.26E+52 | 0.000691 |
| cg14492347 | 2.38519E+19 | 152826024.6 | 3.72E+30 | 0.000691 |
| cg11011602 | 24889601112 | 24519.66075 | 2.52651E+16 | 0.000693 |
| cg02265318 | 595999.281 | 274.3077514 | 1294951167 | 0.000694 |
| cg27534796 | 5.54E+27 | 5.12723E+11 | 6.00E+43 | 0.000695 |
| cg09339301 | 1.29199E+14 | 902575.4631 | 1.85E+22 | 0.000696 |
| cg06434451 | 217.8297373 | 9.68002777 | 4901.824209 | 0.000702 |
| cg21584983 | 4.32E+21 | 1316723376 | 1.42E+34 | 0.000704 |
| cg04223442 | 1.71528E+18 | 48453919.86 | 6.07E+28 | 0.000704 |
| cg19591588 | 8.97735E+15 | 5281191.634 | 1.53E+25 | 0.000705 |
| cg04826422 | 4.7751E+12 | 217688.6425 | 1.05E+20 | 0.000712 |
| cg11454415 | 1.74E+20 | 327446974.5 | 9.30E+31 | 0.000717 |
| cg01333788 | 47.0292319 | 5.050981288 | 437.8849429 | 0.000718 |
| cg17498523 | 3.86481E+16 | 9217148.273 | 1.62E+26 | 0.000729 |
| cg18607470 | 4.69E+28 | 1.08691E+12 | 2.02E+45 | 0.00073 |
| cg03524308 | 2.66706E+11 | 62227.06062 | 1.1431E+18 | 0.000734 |
| cg15990015 | 22047518.22 | 1203.987224 | 4.03736E+11 | 0.000734 |
| cg23121547 | 2747621.892 | 500.9770786 | 15069404135 | 0.000738 |
| cg11453347 | 3.51361E+13 | 471106.7662 | 2.62E+21 | 0.000745 |
| cg16907167 | 9.65922E+13 | 717182.2017 | 1.30E+22 | 0.000747 |
| cg24220122 | 3099451105 | 9331.589412 | 1.02947E+15 | 0.000754 |
| cg07516225 | 4.70554E+14 | 1366785.651 | 1.62E+23 | 0.000755 |
| cg14058345 | 3.41E+27 | 3.25977E+11 | 3.57E+43 | 0.000756 |
| cg18579327 | 3.50723E+12 | 172992.4751 | 7.11052E+19 | 0.000765 |
| cg19254558 | 3.35595E+14 | 1148961.39 | 9.80E+22 | 0.000771 |
| cg05986149 | 5.49581E+12 | 203621.5469 | 1.48E+20 | 0.000779 |
| cg21546057 | 1.8169E+13 | 327211.3934 | 1.01E+21 | 0.000792 |
| cg14694232 | 1.83E+23 | 4673470181 | 7.18E+36 | 0.000796 |
| cg02975259 | 4.8628E+17 | 22376016.51 | 1.06E+28 | 0.000798 |
| cg10869069 | 9.75E+25 | 61534205444 | 1.54E+41 | 0.000805 |
| cg25017304 | 9.65499E+13 | 634757.5134 | 1.47E+22 | 0.000808 |
| cg14711016 | 78650181889 | 32823.67952 | 1.88457E+17 | 0.000816 |
| cg26446827 | 390055352.6 | 3629.580449 | 4.19176E+13 | 0.000818 |
| cg01809579 | 6.18505E+16 | 8819390.525 | 4.34E+26 | 0.00083 |
| cg02686769 | 1.02E+26 | 56821230493 | 1.84E+41 | 0.000833 |
| cg12657853 | 1.96E+20 | 238294449.6 | 1.61E+32 | 0.000844 |
| cg27657283 | 9.81151E+17 | 26475586.07 | 3.64E+28 | 0.000848 |
| cg07246225 | 130089.3937 | 128.6367129 | 131558479.5 | 0.00085 |
| cg18211447 | 3.10268E+12 | 141027.7439 | 6.82604E+19 | 0.000855 |
| cg14407667 | 6.36021E+13 | 477188.985 | 8.48E+21 | 0.000869 |
| cg23104823 | 1.73104E+11 | 41853.28241 | 7.15954E+17 | 0.000872 |
| cg12635664 | 9.56841E+13 | 560117.5544 | 1.63E+22 | 0.000873 |
| cg19826026 | 90.78276546 | 6.38179335 | 1291.409805 | 0.000874 |
| cg05919661 | 2.15723E+17 | 13223499.08 | 3.52E+27 | 0.000879 |
| cg07020962 | 5.03E+28 | 6.12008E+11 | 4.13E+45 | 0.000882 |
| cg10432177 | 4.17053E+18 | 43935409.93 | 3.96E+29 | 0.000886 |
| cg11780382 | 7.8735E+18 | 56061682.58 | 1.11E+30 | 0.000893 |
| cg21341271 | 8.53242E+15 | 3392733.142 | 2.15E+25 | 0.000895 |
| cg13688966 | 38.50151043 | 4.455531336 | 332.7024756 | 0.000907 |
| cg06420088 | 1.14926E+16 | 3725660.733 | 3.55E+25 | 0.000909 |
| cg01522975 | 6.1008E+11 | 66170.97191 | 5.62478E+18 | 0.000911 |
| cg20419410 | 2.66E+81 | 1.99E+33 | 3.54E+129 | 0.000913 |
| cg10266188 | 88692899300 | 29982.04422 | 2.62371E+17 | 0.000913 |
| cg08147886 | 21544968838 | 16677.30423 | 2.78334E+16 | 0.000919 |
| cg09144196 | 2.13154E+11 | 42308.87619 | 1.07388E+18 | 0.000923 |
| cg16688376 | 87441386 | 1750.443401 | 4.36803E+12 | 0.000924 |
| cg20251973 | 2.11E+39 | 1.12062E+16 | 3.97E+62 | 0.000928 |
| cg07453618 | 1.14E+71 | 9.95E+28 | 1.31E+113 | 0.000929 |
| cg03442064 | 4.66097E+11 | 57618.58216 | 3.77043E+18 | 0.000931 |
| cg27575501 | 1.2012E+14 | 547983.5761 | 2.63E+22 | 0.000938 |
| cg08618113 | 1.30E+25 | 16332090752 | 1.04E+40 | 0.000956 |
| cg24692716 | 2237801584 | 6323.433531 | 7.91936E+14 | 0.000958 |
| cg21774863 | 1.75E+21 | 427807102.4 | 7.20E+33 | 0.000963 |
| cg14972143 | 236841.8518 | 152.4659549 | 367912054.6 | 0.000964 |
| cg12623088 | 82842570.66 | 1646.179337 | 4.16898E+12 | 0.000964 |
| cg01407797 | 89442676263 | 27878.09012 | 2.86963E+17 | 0.00097 |
| cg09378691 | 58692845943 | 23393.93117 | 1.47254E+17 | 0.000973 |
| cg09747578 | 723458.2356 | 237.4199713 | 2204497860 | 0.000979 |
| cg24679082 | 8.49601E+15 | 2822938.51 | 2.56E+25 | 0.000988 |
| cg14029663 | 8514.390034 | 39.02240323 | 1857774.808 | 0.000989 |
| cg17797182 | 2.41E+45 | 2.31689E+18 | 2.50E+72 | 0.000994 |
| cg04154812 | 1.6395E+13 | 222263.9722 | 1.21E+21 | 0.000995 |
