# Supplementary material for: DNA methylation-based classification and identification of bladder cancer prognosis-associated subgroups
Source: Cancer Cell Int. 2020 Jun 17;20:255. doi: 10.1186/s12935-020-01345-1 (PMC7302382; doi:10.1186/s12935-020-01345-1)
Supplement: Supplementary file 2 — Additional file 2: Table S2. 557 corresponding promotor genes. [file 12935_2020_1345_MOESM2_ESM.docx]

**Additional file 2: Table S2 557 corresponding promotor genes.**

| Genes |
| --- |
| AC004819.1  AC004854.4  AC006538.4  AC007879.5  AC011290.4  AC068831.10  AC068831.16  AC144652.1  ACBD3  ACO2  ACSL3  ACTR1A  ADRM1  AGBL5  AGBL5-AS1  AGO4  AIDA  AIG1  AKAP9  ALDH18A1  ANXA7  AP1G1  AP2A2  ARFIP2  ARFRP1  ARHGDIB  ARHGEF11  ARL13B  ASCC3  ASF1B  ASPM  ATMIN  ATP6V1F  ATRIP  BCCIP  BIK  BLOC1S4  BLOC1S6  BRCA1  BRIP1  BROX  BSDC1  BUB1  C11orf54  C16orf70  C16orf87  C19orf24  C4orf29  C6orf1  C6orf62  C7orf25  C9orf43  C9orf64  CACNA1G  CACNA1G-AS1  CAHM  CALCOCO2  CAP1  CAPZA1  CASP7  CASP8AP2  CCDC117  CCDC121  CCDC85B  CD164  CDC20  CDC26  CDC45  CDK12  CDK2  CDKL1  CDKL3  CDKN1B  CENPO  CENPQ  CEP250  CEP72  CFAP36  CHMP2A  CHP1  CITED2  CLDN7  CNOT7  COA4  COX10  COX10-AS1  COX15  COX7A2L  CREB3  CTB-129P6.4  CTB-55O6.12  CTC-543D15.8  CTD-2105E13.16  CTD-2506P8.6  CTD-2574D22.6  CTD-2630F21.1  CTD-3028N15.1  CTD-3222D19.2  CUTA  CUTC  CYP46A1  DAAM1  DCAF6  DCLRE1C  DDIT3  DDX1  DDX28  DDX39A  DDX59  DGCR8  DIMT1  DIRC2  DIS3L  DLL1  DMAP1  CDH13  DNAJB2  DNAJC18  DNAJC27  DNAJC27-AS1  DNM2  DPY19L3  DSP  DTL  DUS2  DUSP16  DUSP6  DUSP8  DYNLL1  DYNLL1-AS1  EBAG9  ECSIT  EFCAB5  EGR1  EIF4E  ENC1  EPB41  EPS15  ERBB2  ERH  ERICH1  ETV4  EXD1  EXOC7  EXOC8  FADS2  FAM117A  FAM120B  FAM213B  FAM214B  FAM216A  FAM220A  FAM60A  FASN  FBXO15  FBXO28  FEN1  FGF8  FGF9  FIBP  FKBP14  FLAD1  FOSB  FOXP4-AS1  GAS2  GBAS  GDF5  GFOD1  GJB5  GJB6  GNB2L1  GPCPD1  GPN1  GPN2  GPN3  GPX4  GRN  GS1-279B7.2  GTF2A1  GTF2H5  H2AFV  HDGF  HELB  HEXB  HIBADH  HILPDA  HIP1  HIPK1  HIPK1-AS1  HNRNPK  HOMER1  HPS4  HSF2  HSPA5  HSPBAP1  IDH1  IDI1  IMMT  IMPDH1  INSIG1  INTS7  IPO11  IPO7  IPO9-AS1  IRF2BPL  ITGA2  IVNS1ABP  KIAA0513  KIAA0895  KIF13B  KLF7  KRTAP5-AS1  KXD1  LACE1  LARS2  LIMD1-AS1  LINC01562  LMAN1  LMBR1L  LNP1  LNPEP  LNX2  LRRC41  LRRC59  MAGEF1  MAPKAP1  MAPRE2  MARCKSL1  MBD6  MBTPS1  MCAT  MCC  MCM8  MDM4  MDN1  MEAF6  MED15  MED22  MED26  MEIS2  MEMO1  METTL21B  MFSD5  MFSD8  MIR3656  MIR616  MIR638  MIR6853  MIR7845  MIR92B  MOK  MORN1  MPC2  MRPL36  MRPL47  MRPL57  MRPS24  MRPS31  MRPS35  MTERF1  MTERF3  MUC1  MUT  MYO7A  NABP2  NAP1L1  NARFL  NARS2  NASP  NBR2  NCAPD3  NCK1  NCK1-AS1  NCOR1  NDUFA4L2  NDUFAF4  NDUFAF6  NDUFB5  NEO1  NIPSNAP3A  NME9  NOC3L  NUDT9  NUMBL  NXPH4  OCIAD1  OLFM4  OVOL2  P3H1  PAAF1  PAPD4  PAPD7  PARL  PARP4  PCGF1  PDCD5  PDCD7  PDPK1  PEX13  PGAP3  PGM2  PHF12  PHF5A  PHLDA2  PIGO  PIGV  PIK3CA  PIKFYVE  PIPOX  PLEKHA8  PLK2  PMEL  POLE  POLE3  POLR1D  POLR2E  PPA1  PPOX  PPP1R11  PPP1R21  PPP6C  PRDX5  PRKAG1  PRPF39  PRPF4  PRPF4B  PSIP1  PTDSS1  PTGES3  PTRH2  PTRHD1  PURA  PURG  PUS10  PXMP2  QKI  QRSL1  RAB28  RAB3GAP1  RBBP6  RBM34  RDH14  RER1  RFC4  RFX5  RHOA  RMI1  RNF11  RNF138  RNF41  RNU6-510P  RP11-1060J15.4  RP11-1260E13.2  RP11-126K1.6  RP11-155G14.6  RP11-483P21.2  RP11-180M15.7  RP11-182N22.8  RP11-191L17.1  RP11-196G11.1  RP11-212P7.3  RP11-216B9.6  RP11-247L20.3  RP11-24C3.2  RP11-27K13.3  RP11-299G20.2  RP11-301O19.1  RP11-303E16.3  RP11-309L24.2  RP11-310P5.1  RP11-360P21.2  RP11-386G11.10  RP11-40H20.4  RP11-421M1.8  RP11-425D10.10  RP11-46F15.2  RP11-505K9.1  RP11-535A19.2  RP11-53I6.3  RP11-554J4.1  RP11-573D15.3  RP11-574K11.24  RP11-637O19.3  RP11-653J6.1  RP11-65N13.8  RP11-745C15.2  RP11-96O20.4  RP11-96O20.5  RP11-973D8.4  RP13-672B3.2  RP1-4G17.5  RP1-92O14.3  RP3-406P24.3  RP3-467N11.1  RP3-512B11.3  RP4-583P15.15  RP5-864K19.4  RPL10A  RPL29  RPL7A  RPL7L1  RPS10  RPS10-NUDT3  RPS16  RPS25  RPSAP31  RRAGC  RSPO3  RSRC1  RTCA  RTCA-AS1  RTN4IP1  SACM1L  SAR1A  SCCPDH  SCRIB  SDC1  SEC24C  SEC61G  SEMA5B  SENP7  SERAC1  SF3A3  SFSWAP  SFTA2  SGSM2  SH2B1  SIRT7  SKA3  SLC10A2  SLC18B1  SLC20A2  SLC25A17  SLC25A44  SLC30A5  SLC33A1  SLC39A3  SLC39A9  SMAD1  SMEK2  SMIM19  SNORD24  SNORD95  SNRPA1  SNRPD1  SP4  SPRTN  SPTAN1  SPTSSA  SRRD  SSU72  ST13  ST7L  STOML2  SUFU  SUPT5H  SUV420H2  TAF1D  TAF5  TAGLN2  TARBP1  TBC1D19  TBCA  TCP11L2  TCTA  TGOLN2  TGS1  TIMM10B  TIMM21  TIMM44  TLN1  TM4SF4  TMEM132A  TMEM14B  TMEM161B  TMEM161B-AS1  TMEM258  TMEM68  TMX1  TOMM34  TOMM40  TOMM70A  TOR3A  TPRG1L  TRA2A  TRA2B  TRAPPC4  TREX1  TRIM26  TRMT112  TRMT6  TSC22D2  TSFM  TSR1  TTF2  TTI2  TUBA1B  TUBA4A  TUBGCP6  TUFM  TXLNA  U2AF2  UBXN1  UFD1L  UHMK1  UQCRH  UROS  USP30  UVRAG  VCPKMT  VKORC1  VMP1  VPS26B  VPS33B  VPS37A  VPS45  VPS9D1  WDR37  WDR6  WDTC1  WIPI2  WRN  WWP2  XPNPEP3  XPO1  YAE1D1  YIPF4  YPEL1  YWHAH  ZBTB2  ZCCHC11  ZEB2  ZEB2_AS1_3  ZEB2_AS1_4  ZEB2-AS1  ZGPAT  ZMYND11  ZNF133  ZNF148  ZNF273  ZNF276  ZNF322  ZNF335  ZNF426  ZNF428  ZNF467  ZNF550  ZNF566  ZNF585A  ZNF592  ZNF646  ZNF668  ZNF672  ZNF689  ZNF718  ZNF770  ZSCAN21 |
