# Supplementary figures and images for: DNA methylation-based classification and identification of bladder cancer prognosis-associated subgroups
Source: Cancer Cell Int. 2020 Jun 17;20:255. doi: 10.1186/s12935-020-01345-1 (PMC7302382; doi:10.1186/s12935-020-01345-1)

A

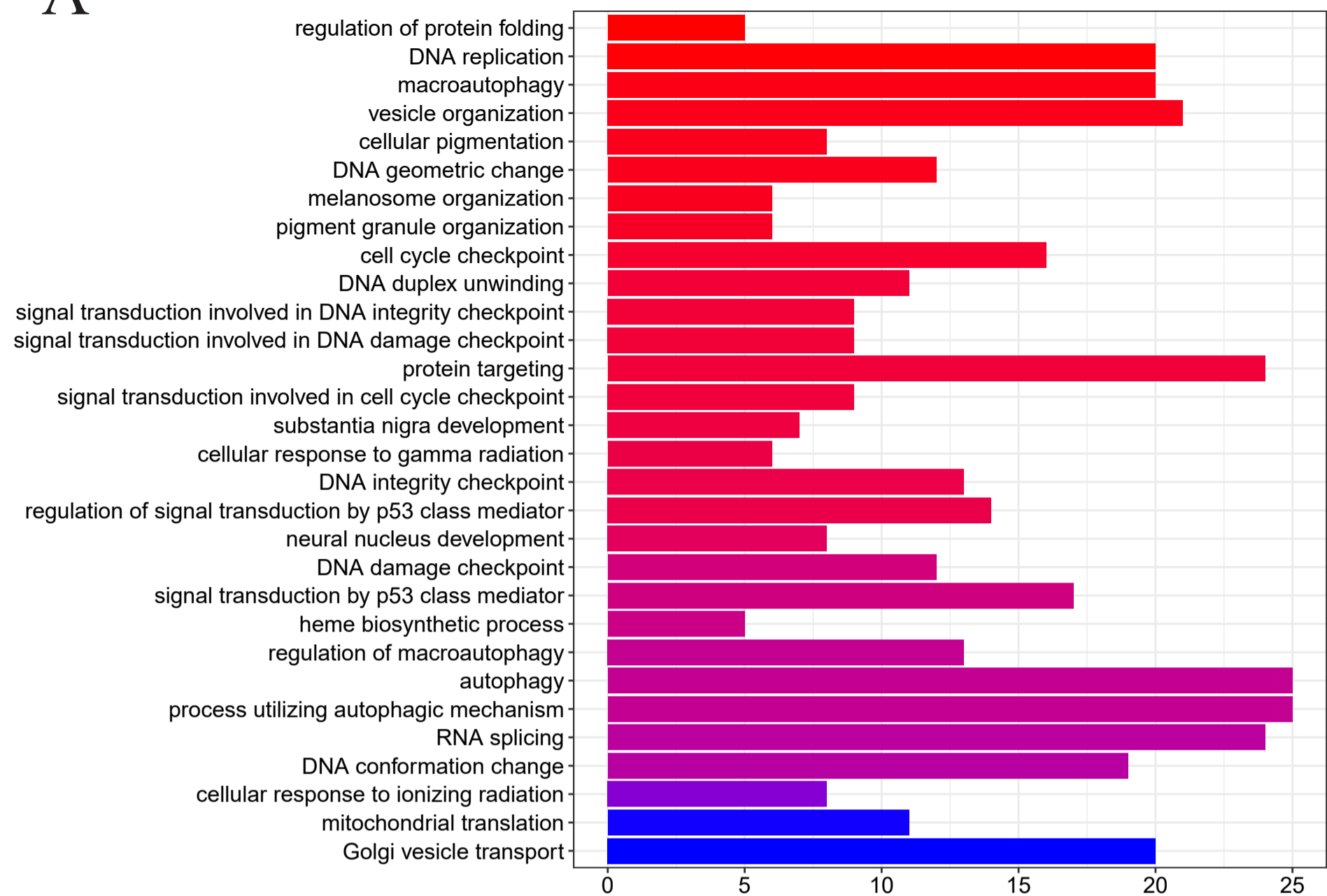

B

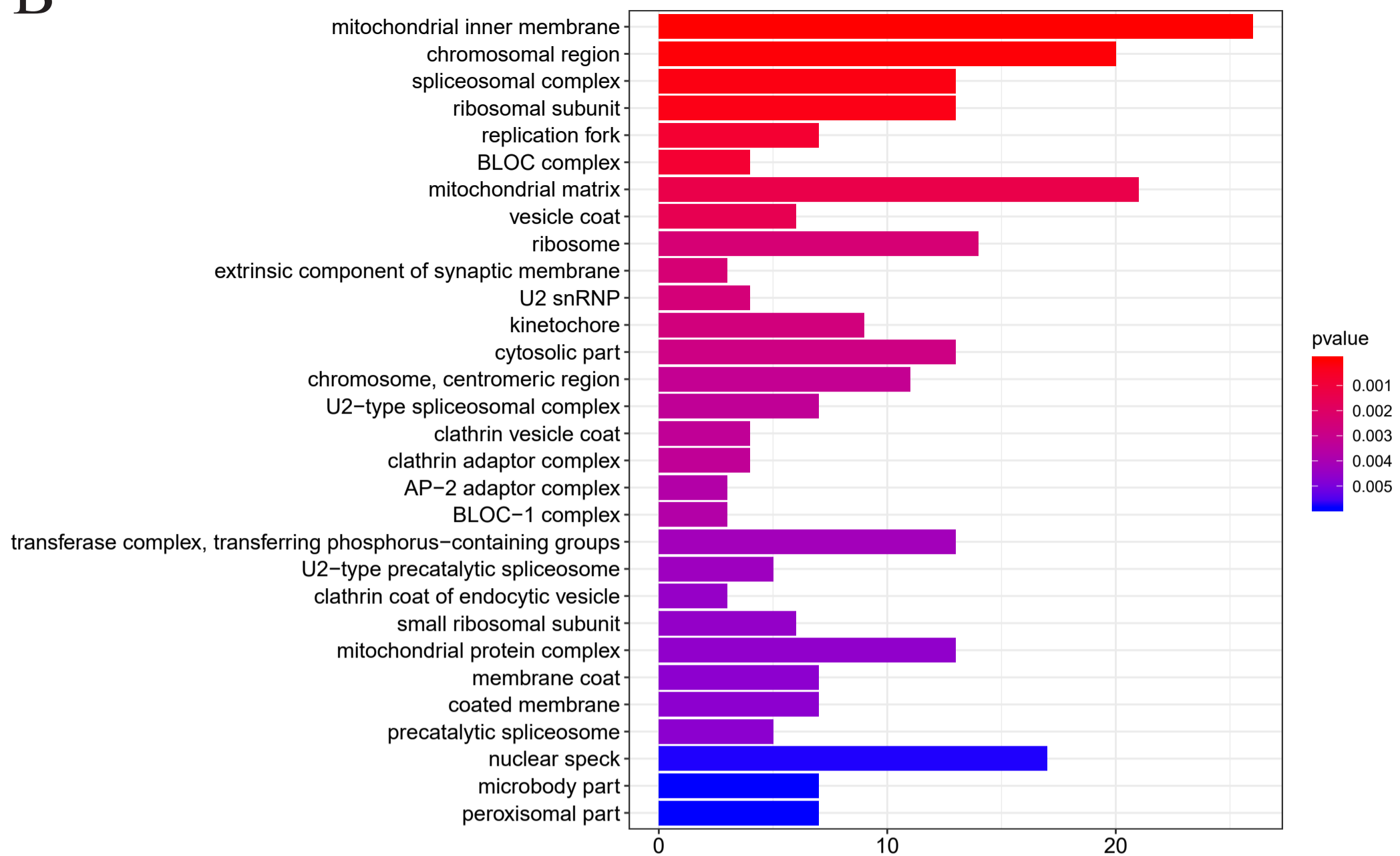

C

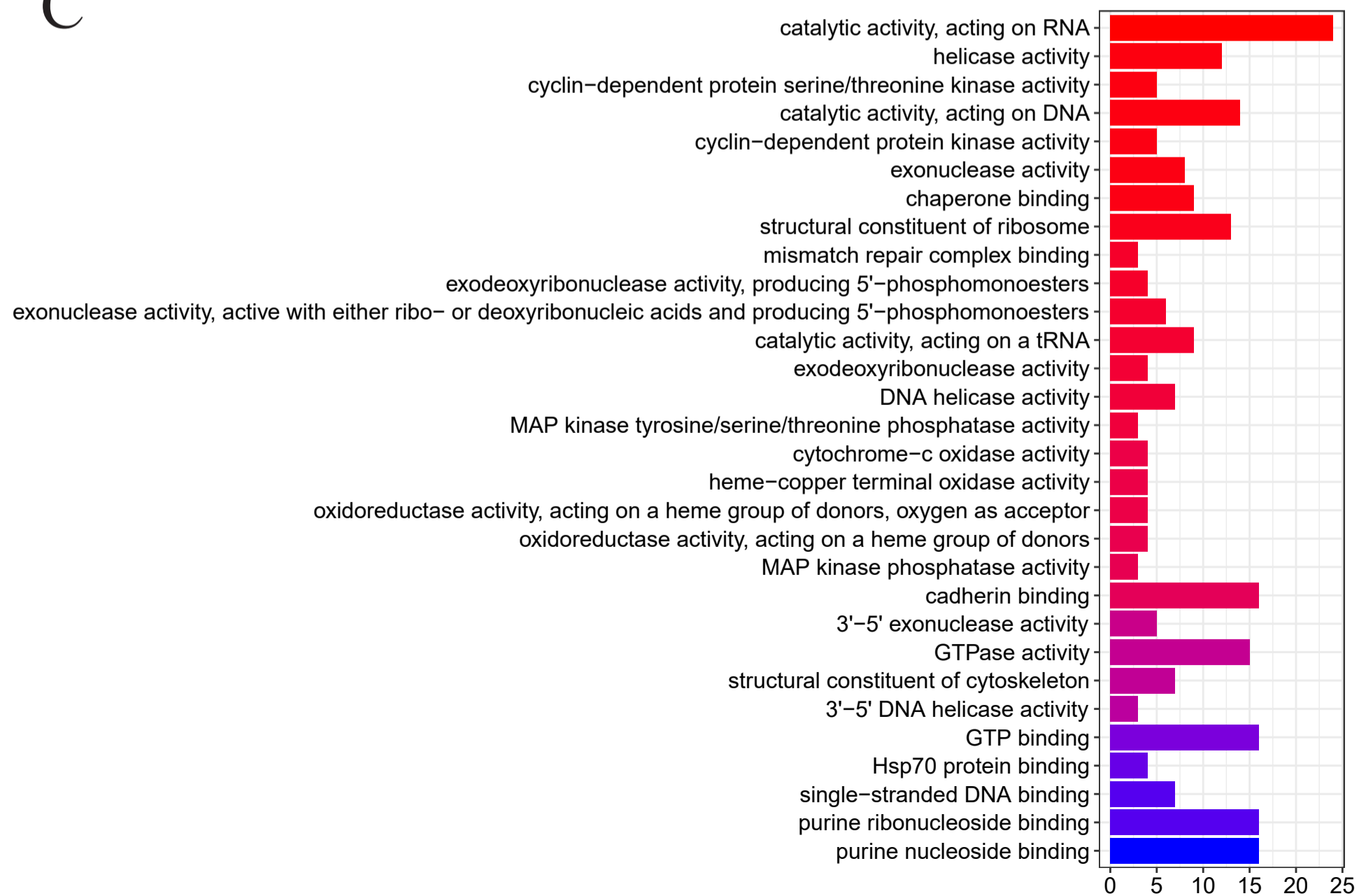

D

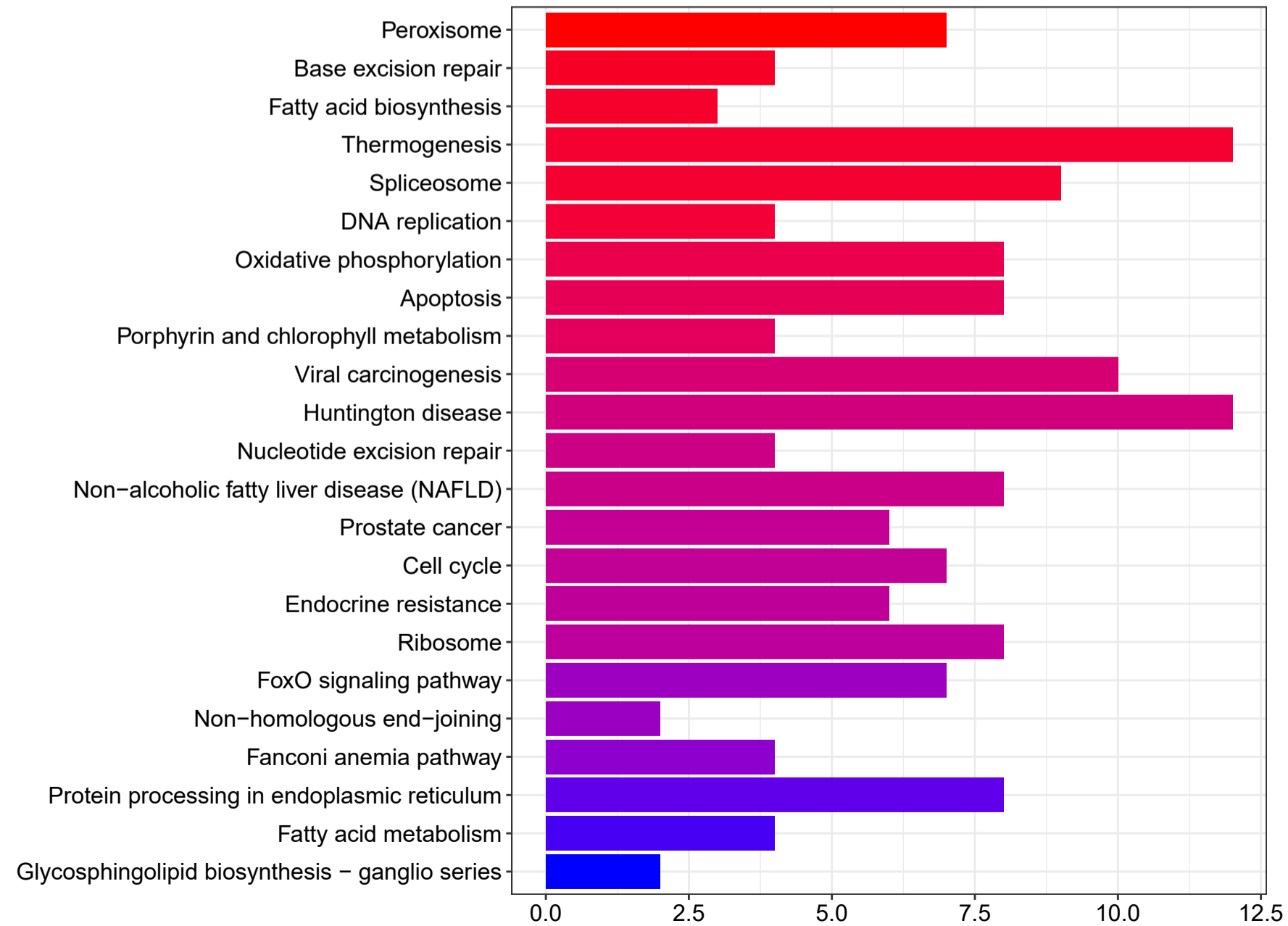

Supplement: Supplementary file 3 — Additional file 3: Figure S1. Enriched GO terms and KEGG pathways. a GO terms for biological process. b GO terms for cellular component. c GO terms for molecular function. d KEGG pathways. [file 12935_2020_1345_MOESM3_ESM.pdf]

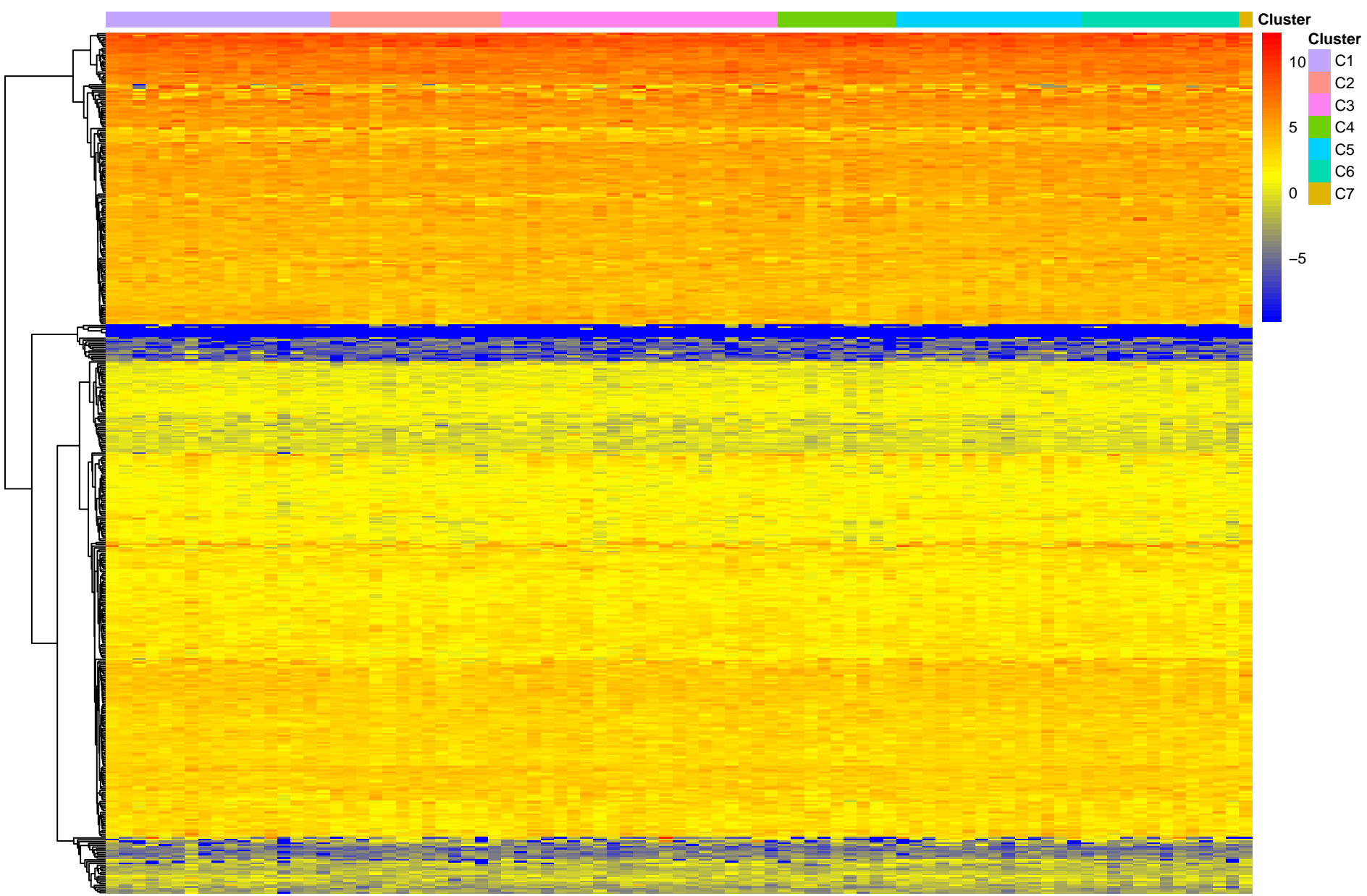

Supplement: Supplementary file 4 — Additional file 4: Figure S2. Heatmap of annotated genes associated with the 456 CpGs. [file 12935_2020_1345_MOESM4_ESM.pdf]

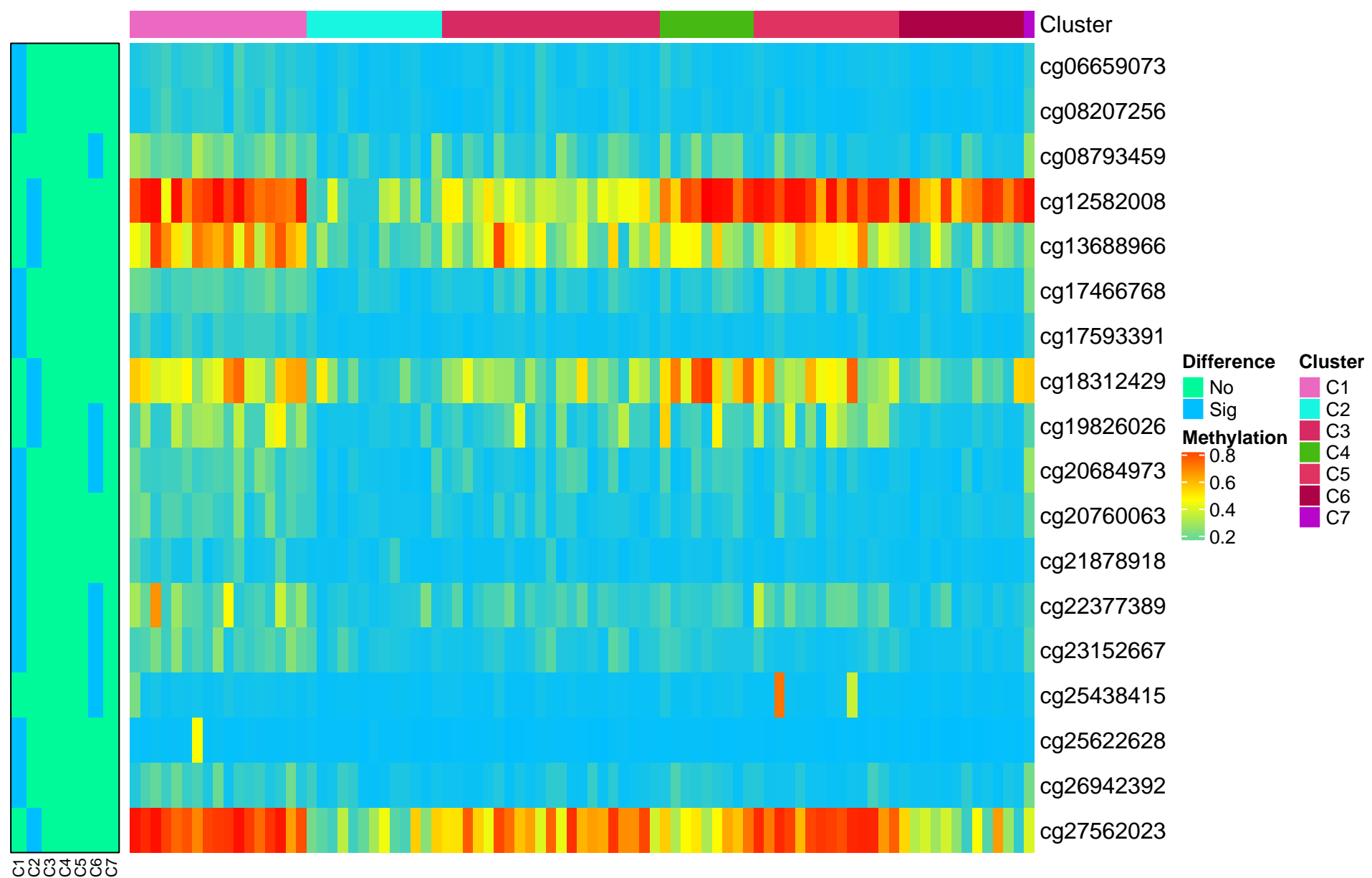

Supplement: Supplementary file 5 — Additional file 5: Figure S3. Specific methylation of CpGs for each DNA methylation cluster. Specific CpGs are shown for each DNA methylation prognostic subtype. Red and blue represent hyper- and hypomethylated CpGs, respectively. [file 12935_2020_1345_MOESM5_ESM.pdf]
