# Supplementary material for: DNA methylation-based classification and identification of bladder cancer prognosis-associated subgroups
Source: Cancer Cell Int. 2020 Jun 17;20:255. doi: 10.1186/s12935-020-01345-1 (PMC7302382; doi:10.1186/s12935-020-01345-1)
Supplement: Supplementary file 6 — Additional file 6: Table S3. The distribution of samples and cluster-specific CpG sites based on 7 prognosis subgroups in the training groups [file 12935_2020_1345_MOESM6_ESM.docx]

**Additional file 6: Table S3**. The distribution of samples and cluster-specific CpG sites based on 7 prognosis subgroups in the training groups

|  | Samples | cluster-specific CpG sites |
| --- | --- | --- |
| C1 | 17 | 11 (cg06659073, cg08207256, cg174466768, cgcg17593391, cg20684973, cg20760063, cg21878918, cg22377389, cg23152667, cg25622628, cg26942392 ) |
| C2 | 13 | 5 (cg12582008, cg13688966, cg18312429, cg19826026, cg27562023) |
| C3 | 21 | 0 |
| C4 | 9 | 0 |
| C5 | 14 | 0 |
| C6 | 12 | 6 (cg08793459, cg19826026, cg20684973, cg22377389, cg23152667, cg25438415) |
| C7 | 1 | 0 |
